# Comprehensive Extraction of Shrimp Head Lipids and Peptides from *Litopenaeus vannamei*: Evaluation of Neuroprotective Potential and Constituent Analysis

**DOI:** 10.3390/foods15111999

**Published:** 2026-06-03

**Authors:** Jiawen Zhao, Longjian Zhou, Yayue Liu, Zhiyou Yang, Fangfang Ban, Yi Zhang

**Affiliations:** 1 Guangdong Provincial Key Laboratory of Aquatic Product Processing and Safety, Guangdong Provincial Engineering Laboratory for Marine Biological Products, Shenzhen Institute of Guangdong Ocean University, Zhanjiang Municipal Key Laboratory of Marine Drugs and Nutrition for Brain Health, Research Institute for Marine Drugs and Nutrition, College of Food Science and Technology, Guangdong Ocean University, Zhanjiang 524088, China; 19878308689@163.com (J.Z.); zhoulongjian@gdou.edu.cn (L.Z.); liuyayue@gdou.edu.cn (Y.L.); zyyang@gdou.edu.cn (Z.Y.); 1125135@gdou.edu.cn (F.B.); 2 Southern Marine Science and Engineering Guangdong Laboratory (Zhanjiang), Zhanjiang 524088, China; 3 Collaborative Innovation Center of Seafood Deep Processing, Dalian Polytechnic University, Dalian 116034, China

**Keywords:** *Litopenaeus vannamei*, lipids, peptides, antioxidant activity, neuroprotection, anti-neuroinflammation

## Abstract

The increasing prevalence of Alzheimer’s disease has created a substantial and urgent need for brain-healthy functional foods. The processing of Pacific white shrimp (*Litopenaeus vannamei*) generates considerable amounts of head waste, which is rich in bioactive compounds, including lipids and peptides, holding great promise for the development of nutraceuticals to support human brain health. However, traditional extraction methods are time-consuming and inefficient in fully utilizing these compounds. This study aimed to explore the functional properties of these shrimp head-derived ingredients using “one-step” three-phase partitioning (TPP) followed by successive proteolysis. The extracted polar lipid (PL-SH), protein (P-SH) and proteolytic peptidic product (Pep-SH) from shrimp heads were screened for their antioxidant, neuroprotective, and anti-neuroinflammatory activities. Antioxidant activities were evaluated using 2,2-diphenyl-1-picrylhydrazyl (DPPH), 2,2′-azino-bis(3-ethylbenzothiazoline-6-sulfonic acid) (ABTS^+^), and hydroxyl free radical scavenging assays, all of which revealed strong antioxidant potential for all three products. Neuroprotective activities were assessed using HT-22 mouse hippocampal neuronal cells challenged with Aβ_25−35_, and anti-neuroinflammatory activities were evaluated using BV-2 microglial cells stimulated with lipopolysaccharide (LPS). The results suggested that both PL-SH and Pep-SH exerted protective effects against Aβ_25−35_-induced cell damage under the tested conditions, and PL-SH also reduced nitric oxide (NO) production induced by LPS, indicating potential anti-neuroinflammatory activity. However, further studies with additional biomarkers (e.g., ROS, apoptosis markers, and cytokines) are required to confirm these effects. The lipid composition of PL-SH was further characterized by thin-layer chromatography and LC-MS/MS-based lipidomics, revealing various classes of phospholipids. Furthermore, analysis of the molecular weight distribution and sequences of peptides in Pep-SH revealed peptide sizes ranging from 70 to 1700 Da and a high degree of homology to known antioxidant and neuroprotective peptide sequences. These findings suggest that lipids and peptides from Pacific white shrimp heads possess valuable functional properties, supporting their potential use in the development of functional foods for neuroprotection and anti-neuroinflammation.

## 1. Introduction

Alzheimer’s disease (AD) is the most common form of dementia, characterized by progressive memory loss, cognitive dysfunction, and behavioral changes. The complex pathogenesis of AD remains incompletely understood. The mainstream amyloid cascade hypothesis posits that AD pathogenesis is driven by the accumulation of Aβ, which promotes the formation of amyloid plaques in the brain [1]. Moreover, Aβ and amyloid plaques can induce inflammation, tau hyperphosphorylation, oxidative damage, and lipid peroxidation through various secondary mechanisms [2,3,4]. Conversely, these pathological processes can further promote Aβ aggregation and deposition, thereby exacerbating AD pathology. Today, AD is the fourth leading cause of death worldwide, following heart disease, cancer, and stroke [5]. For this typical neurodegenerative disease, early intervention is believed to slow cognitive decline more effectively than late-stage treatment [6,7,8]. Therefore, developing beneficial functional foods or nutraceuticals to address this health crisis is of great importance.

Pacific white shrimp (PWS) (*Litopenaeus vannamei*) is native to the warm waters along the Pacific coast of Central and South America. This shrimp is rich in high-quality protein, low in fat, and contains various minerals (e.g., calcium, magnesium, zinc) and vitamins (e.g., A, D, E), making it a nutritious seafood product [9]. It is particularly known for its fast growth, strong stress resistance, high environmental adaptability, and thin shell with thick flesh, which endow it with significant economic value [10]. Today, it has become one of the three most farmed shrimp species globally and the most widely farmed species in southern China [11], with a national production of 2.2384 million tons in 2024 [12].

The flourishing PWS processing industry generates tremendous amounts of waste, most of which consist of shrimp heads rich in bioactive compounds, including chitin, proteins, lipids, carotenoids, and minerals [13]. The shrimp head contains abundant proteins, with a crude protein content of 6.38% [14]. Peptides derived from PWS heads have been shown to enhance immunity by promoting lymphocyte proliferation, alleviating thymus and spleen atrophy, and improving the phagocytic capacity of immune cells [15]. The shrimp head also contains a variety of lipids, especially phospholipids, such as phosphatidylcholine (PC), phosphatidylethanolamine (PE), phosphatidylinositol (PI), phosphatidylserine (PS), and sphingomyelin (SM). These phospholipids not only possess high nutritional value but also play a positive role in preventing cardiovascular diseases, cancer, and neurological disorders [16]. However, the simultaneous extraction of both protein and lipids is challenging due to their widely differing solubilities. Currently, most extraction processes still rely on traditional acid–alkali or solvent-based methods, which yield only a single product and significantly increase the environmental burden due to the use of chemical reagents [14].

Three-phase partitioning (TPP) is a non-chromatographic bioseparation technique [17]. By mixing a crude extract with solid salts and organic solvents, a three-phase system is formed through salting-out, isoelectric precipitation, and co-solvent precipitation [18]. TPP was first introduced in the 1970s for protein extraction. Through continuous development and innovation, TPP is no longer limited to proteins but has been widely applied to the extraction of various components, such as lipids and polysaccharides, offering a simple, rapid, efficient, and low-cost approach [19].

Previous studies have reported that shrimp head-derived peptides exhibit antioxidant, immunomodulatory, and antihypertensive activities [15]. Likewise, shrimp head phospholipids, particularly phosphatidylcholine and phosphatidylethanolamine, have been shown to possess cardiovascular protective effects [16]. However, the simultaneous preparation of both protein and peptides from shrimp head and the neuroprotective and anti-neuroinflammatory potential of these compounds remains largely unexplored. This study aimed to extract polar lipids, proteins, and peptide hydrolysates from shrimp heads using a one-step three-phase partitioning (TPP) approach followed by proteolysis, and to systematically evaluate their neuroprotective and anti-neuroinflammatory activities. This integrated strategy maximizes the valorization of shrimp head waste and provides a green and efficient alternative to conventional single-product extraction methods.

## 2. Materials and Methods

### 2.1. Experimental Materials and Cell Strain Sources

Pacific white shrimp (*Litopenaeus vannamei*) heads: purchased from Zhanjiang Guolian Aquatic Development Co., Ltd., Zhanjiang, China. Mouse hippocampal neuron cells (HT-22): obtained from the Cell Resource Center at the Shanghai Institute of Biological Sciences, Chinese Academy of Sciences (GNM47, Shanghai, China). Mouse microglial cells (BV-2): obtained from the Chinese Typical Culture Collection Center at Wuhan University (GDC0311, Wuhan, Hubei, China).

### 2.2. Main Instruments and Reagents

Agilent 1260 InfinityII (Agilent Technologies, Santa Clara, CA, USA); FD-1D-50 freeze dryer (Beijing Boyikang Experimental Instruments Co., Ltd., Beijing, China); CKX41-A32PH inverted microscope (Olympus Corporation, Tokyo, Japan); IC 1000 cell counter (Shanghai Ruiyu Biotechnology Co., Ltd., Shanghai, China); CI-191C CO_2_ incubator (Guangzhou Yuwei Biotechnology Instrument Co., Ltd., Guangzhou, China); H180R centrifuge (Hunan Xiangyi Experimental Instruments Co., Ltd., Changsha, Hunan, China); N-1001 rotary evaporator (Tokyo Rikakikai Co., Ltd., Tokyo, Japan); Biotek Epoch 2 full-spectrum microplate reader (BioTek Instruments, Inc., Winooski, VT, USA); Acquity UHPLC-DAD-Xevo G2-XS Q-Tof (Waters Corporation, Milford, MA, USA); Easy-nLC 1200/QExactive (Thermo Fisher Scientific, Waltham, MA, USA).

3-(4,5-dimethylthiazol-2-yl)-2,5-diphenyltetrazolium bromide (MTT) (Shanghai Yuanye Bio-Technology Co., Ltd., Shanghai, China); lipopolysaccharide (LPS) (Sigma-Aldrich, St. Louis, MO, USA); 2,2-diphenyl-1-picrylhydrazyl (DPPH) (Sigma-Aldrich, St. Louis, MO, USA); 2,2′-azino-bis(3-ethylbenzothiazoline-6-sulfonic acid) (ABTS^+^) (Shanghai Yuanye Bio-Technology Co., Ltd., Shanghai, China); fetal bovine serum (FBS) (Zeta Corporation, Monrovia, CA, USA); Aβ_25−35_ (MCE Company, Monmouth Junction, NJ, USA); Dulbecco’s Modified Eagle Medium (DMEM) (Gibco, Thermo Fisher Scientific, Waltham, MA, USA); chloroform, methanol, n-butanol, and ammonium sulfate (Guangdong Guanghua Technology Co., Ltd., Shantou, China, analytical pure); alkaline protease (Shanghai Yuanye Bio-Technology Co., Ltd., Shanghai, China, 200 U/mg).

### 2.3. Three-Phase Extraction of the Heads of Pacific White Shrimp

The shrimp heads were lyophilized for 24 h and then ground into a powder using a mill. A 30% (*w*/*v*) ammonium sulfate solution was prepared, and the pH was adjusted to approximately 5.0. This solution was then mixed with an equal volume of n-butanol. The shrimp head powder was added to the mixture at a liquid-to-solid ratio of 3:1 (*v*/*w*). The resulting mixture was stirred at room temperature for 30 min and then centrifuged at 4500 r/min for 10 min [20]. After centrifugation, the three layers were separately collected: the top layer (lipids and organic solvents), the middle layer (protein precipitate), and the bottom layer (chitin and water-soluble substances).

### 2.4. Preparation of PL-SH

The first collected fraction was concentrated using a rotary evaporator at 65 °C to remove the organic solvent, yielding a shrimp oil concentrate. After cooling, pre-cooled acetone was added to the concentrate, and the mixture was shaken for extraction. The mixture was then transferred to a centrifuge tube and centrifuged at 8000 r/min for 6 min. After the supernatant was removed, the resulting polar lipid precipitate (PL-SH) was dried and weighed [21].

### 2.5. Preparation of P-SH and Pep-SH

The protein precipitate fraction was collected, and excess lipids were extracted using an appropriate amount of petroleum ether. Subsequently, the material was lyophilized overnight to yield a protein powder (P-SH). Half of the powder was used for enzymatic hydrolysis, while the other half was stored at −80 °C.

For enzymatic hydrolysis, the protein powder was dissolved in water at a mass ratio of 1:12 (powder to water). Alkaline protease (1%, *w*/*v*) was then added, and the pH was adjusted to approximately 9.0. Hydrolysis was carried out at 55 °C for 3 h. The enzyme was then inactivated by heating at 90 °C for 10 min. After inactivation, the sample was centrifuged at 4500 r/min for 10 min, and the supernatant was collected. Finally, the supernatant was lyophilized overnight to obtain the peptide powder (Pep-SH) [15].

### 2.6. Antioxidant Activity Evaluation

Stock solutions of PL-SH (in absolute ethanol), P-SH (in ultrapure water), and Pep-SH (in ultrapure water) were prepared at final concentrations of 0.01, 0.05, 0.15, 0.4, and 1.0 mg/mL. Ascorbic acid solutions at the same concentrations were used as positive controls, and all assays were performed in triplicate.

The DPPH radical scavenging assay was performed as previously described [22]. Briefly, a 0.26 mmol/L DPPH solution was prepared in anhydrous ethanol. A total of 50 μL of the sample solution was mixed with 50 μL of the DPPH solution. After incubation in the dark at room temperature for 30 min, the absorbance was measured at 517 nm using a microplate reader. The DPPH scavenging capacity was calculated according to the following formula:

(1) $$\mathrm{DPPH}\;\mathrm{radical}\;\mathrm{scavenging}\;\mathrm{rate}\;=\left(1-\frac{A_{1}-A_{2}}{A_{0}}\right)\times 100\%$$

where *A*_1_ is the absorbance after the reaction of the sample solution with the DPPH solution; *A*_2_ is the control group, with anhydrous ethanol replacing the DPPH solution; *A*_0_ is the blank group, with the sample solvent replacing the sample solution.

The ABTS^+^ radical scavenging activity was determined according to a previously described protocol [23]. Briefly, a stock solution was prepared by mixing 7 mM ABTS with 2.45 mM potassium persulfate at a 1:1 ratio (*v*/*v*). The mixture was incubated in the dark at room temperature for 12–18 h to generate ABTS^+^ radicals. The resulting solution was then diluted to an absorbance of 0.7 ± 0.02 at 734 nm, and this dilution was used as the ABTS^+^ working solution. In the assay, 150 μL of ABTS^+^ working solution and 50 μL of sample solution were added to each well of a 96-well plate. The mixture was incubated in the dark at room temperature for 5 min, after which the absorbance was recorded at 734 nm. The ABTS^+^ scavenging capacity was calculated according to the following formula:

(2) $${\mathrm{ABTS}}^{+}\;\mathrm{cation}\;\mathrm{radical}\;\mathrm{scavenging}\;\mathrm{rate}\;=\left(1-\frac{B_{1}-B_{2}}{B_{0}}\right)\times 100\%$$

where *B*_1_ is the absorbance after the reaction of the sample solution with the ABTS^+^ working solution; *B*_2_ is the control group, with ultrapure water replacing the ABTS^+^ working solution; *B*_0_ is the blank group, with the sample solvent replacing the sample solution.

The hydroxyl radical scavenging assay was performed as previously described [24]. Briefly, 1 mL of sample, 1 mL of 9 mmol/L FeSO_4_ solution, 1 mL of 9 mmol/L salicylic acid solution (in 70% ethanol), and 1 mL of 9 mmol/L H_2_O_2_ solution were sequentially added to a test tube. After incubation in a water bath at 37 °C for 30 min, the absorbance was measured at 510 nm. The hydroxyl radical scavenging capacity was calculated according to the following formula:

(3) $$\mathrm{Hydroxyl}\;\mathrm{radical}\;\mathrm{clearance}\;\mathrm{rate}\;=\left(1-\frac{C_{1}-C_{2}}{C_{0}}\right)\times 100\%$$

where *C*_1_ is the absorbance after the reaction of the sample solution with the three reagents; *C*_2_ is the control group, with ultrapure water replacing the H_2_O_2_ solution; *C*_0_ is the blank group, with the sample solvent replacing the sample solution.

### 2.7. Neuroprotective Activity Evaluation

Cryopreserved HT-22 mouse hippocampal neuronal cells were thawed and cultured in DMEM supplemented with 10% (*v*/*v*) fetal bovine serum and 1% penicillin-streptomycin (100×) in an incubator at 37 °C with 5% (*v*/*v*) CO_2_. The medium was changed every two days, and the cells were passaged every three days. Cell morphology was observed under a microscope, and cells in the logarithmic growth phase were used for subsequent experiments.

MTT Assay for Determining the Cytotoxicity of PL-SH and Pep-SH on HT-22 Cells: When the cells were in good condition, they were seeded at a density of 1.2 × 10^4^ cells per well in 100 μL volume into a 96-well plate and incubated for 24 h. The experiment was divided into groups: (1) Blank group: DMEM basal medium; (2) Control group: cells + DMEM basal medium; (3) Treatment group: cells + 8 concentration gradients of sample solutions. There were 6 replicates for each group. After incubation for another 24 h, the supernatant was discarded, and 100 μL of MTT was added to each well. After incubation for 4 h, the supernatant was removed again, and 100 μL of analytical grade DMSO was added to each well in the dark. The plate was then shaken on a shaker for 10 min to fully dissolve the formazan. Finally, the absorbance was measured at a wavelength of 490 nm using a microplate reader.

The MTT method was used to assess the cell viability of HT-22 cells damaged by Aβ_25–35_ induction. When the cells grew well, they were seeded at a density of 1.2 × 10^4^ cells per well in 100 μL volume into a 96-well plate and cultured in an incubator for 24 h. The experiment was divided into groups: (1) Blank group: cells + DMEM basal medium; (2) Control group: cells + DMEM basal medium + Aβ_25–35_; (3) Treatment group: cells + 6 concentration gradients of sample solutions + Aβ_25–35_. There were 6 replicates for each group. The experimental method was the same as the MTT method described above to determine the cell survival rate.

### 2.8. Anti-Neuroinflammatory Activity Evaluation

Cryopreserved BV-2 microglial cells were thawed and cultured in DMEM supplemented with 10% (*v*/*v*) fetal bovine serum and 1% penicillin-streptomycin in an incubator at 37 °C with 5% (*v*/*v*) CO_2_. The culture medium was changed every two days, and the cells were passaged every two days. Cell morphology was observed under a microscope, and cells in the logarithmic growth phase were used for subsequent experiments.

Assessment of the Cytotoxicity of PL-SH and Pep-SH on BV-2 Cells Using the CCK-8 Assay: When the cells grew well, they were seeded in a 96-well plate at a density of 1 × 10^4^ cells per well in 100 μL volume and cultured for 24 h. The experimental groups were as follows: (1) Blank group: DMEM basic culture medium; (2) Control group: cells + DMEM basic culture medium; (3) Treatment group: cells + eight concentration gradients of sample solutions (all samples were diluted in DMEM). There were six replicates per group. After culturing for 24 h, 10 μL of CCK-8 solution was added to each well in the dark. After incubation for 1 h, the absorbance was measured at a wavelength of 450 nm using a microplate reader.

The nitric oxide (NO) levels in BV-2 cells induced by lipopolysaccharide (LPS) and uninduced cells were quantitatively detected using the Beyotime NO Assay Kit (Beyotime Biotechnology Co., Ltd., Shanghai, China). When the cells grew well, they were seeded in a 96-well plate at a density of 2 × 10^4^ cells per well in 100 μL volume and cultured in the incubator for 24 h. The experimental groups were as follows: (1) Blank group: cells + DMEM basic culture medium; (2) Control group: cells + DMEM basic culture medium + LPS; (3) Treatment group: cells + six concentration gradients of sample solutions + LPS. Each group was set with six replicates. One hour after adding DMEM basic culture medium and samples to the control and experimental groups, respectively, LPS at a final concentration of 1 μg/mL was added, and the cells were then cultured for 24 h. Afterwards, 30 μL of supernatant was mixed with 30 μL of Reagent I from the kit in the dark. After a 5 min reaction, Reagent II was added, and the mixture was reacted for another 5 min. The absorbance was measured at a wavelength of 540 nm using a microplate reader. The NO standard curve was prepared according to the instructions of the Beyotime NO Assay Kit.

### 2.9. Chemical Analysis of PL-SH

#### 2.9.1. Thin-Layer Chromatographic Analysis

A 5 mg/mL solution of PL-SH in methanol was prepared. A 6 μL aliquot of this solution was spotted onto a normal-phase silica gel plate (Merck KGaA, Darmstadt, Germany) using a capillary tube. Two plates were spotted in parallel. The developing solvent was a mixture of chloroform, methanol, and water (65:22:4, *v*/*v*/*v*), and thin-layer chromatography (TLC) was performed in a glass developing chamber.

After chromatography, the plates were separately stained with phosphomolybdic acid reagent and iodine vapor. The staining results were photographed. The phosphomolybdic acid reagent was prepared from ammonium molybdate, concentrated sulfuric acid, and L-ascorbic acid, as described previously [25]. During staining, a hot air blower was used to apply heat.

#### 2.9.2. Liquid Chromatography-Tandem Mass Spectrometry (LC-MS/MS) Analysis

The analysis was performed on a Waters Acquity UHPLC-DAD-Xevo G2-XS Q-Tof LC-MS system equipped with an ACQUITY UPLC BEH RP18 column (2.1 mm × 50.0 mm, 1.7 μm, Waters, Milford, MA, USA). The PL-SH sample was dissolved in methanol-acetonitrile (4:6, *v*/*v*) at a concentration of 50 μg/mL, and the injection volume was 1 μL. Chromatographic separation was achieved under isocratic conditions using a mixture of methanol (containing 0.1% *v*/*v* formic acid; Solvent A) and acetonitrile (containing 0.1% *v*/*v* formic acid; Solvent B) at a volume ratio of 4:6 (A:B). The flow rate was 0.2 mL/min, and the elution time was 8 min [26]. The mass spectrometry parameters were as follows: mass scanning range, *m*/*z* 100–1700; ionization source, electrospray ionization (ESI) in both positive and negative ion modes; ion source temperature, 120 °C; capillary voltage, 1.6 kV; sampling cone voltage, 40 V; source offset voltage, 80 V; collision energy, 25–55 eV; desolvation temperature, 450 °C; cone gas flow rate, 50 L/h; and desolvation gas flow rate, 800 L/h. The acquired LC-MS/MS data were processed using the Lipidomics function of the MS-DIAL metabolomics analysis software (Version 4.70) for annotation of the main characteristic components [27].

### 2.10. Chemical Analysis of Pep-SH

#### 2.10.1. Determination of Molecular Weight

The determination of molecular weight for peptides was carried out using gel permeation chromatography (GPC) with the following parameters: instrument: Agilent 1260 HPLC; detector: Agilent RID G1362A (Agilent Technologies, Santa Clara, CA, USA); column: Waters Ultrahydrogel (Waters Corporation, Milford, MA, USA) 300 mm × 7.8 mm 500–250–120 A; mobile phase: 0.1 mol/L NaNO_3_ aqueous solution; flow rate: 1 mL/min; temperature: 40 °C; standard: PEG (Polyethylene Glycol).

#### 2.10.2. Peptide Sequencing Analysis of Pep-SH

Reduction and alkylation: exactly 5 mg of Pep-SH was weighed into a 1.5 mL microcentrifuge tube and dissolved in 400 μL of ultrapure water. Then, 1 μL of 1 M dithiothreitol (DTT) solution was added to achieve a final DTT concentration of 10 mM, and the mixture was incubated at 56 °C for 1 h to reduce disulfide bonds. Subsequently, 2 μL of 1 M iodoacetamide (IAM) solution was added to reach a final IAM concentration of 20 mM, and the alkylation was allowed to proceed in the dark at room temperature for 40 min. Thereafter, 1 μL of 1 M DTT solution (final concentration 10 mM) was added to quench any unreacted IAM. Finally, the sample was desalted using an SP2 column.

LC-MS/MS conditions: a trap column (150 μm × 50 mm, 3 μm, Thermo Fisher Scientific, Waltham, MA, USA) packed with ReproSil-Pur 120 C18-AQ 3 μm particles, and an analytical column (150 μm × 170 mm, 1.9 μm, Thermo Fisher Scientific, Waltham, MA, USA) packed with ReproSil-Pur 120 C18-AQ (Dr. Maisch GmbH, Ammerbuch, Germany) 1.9 μm particles. Mobile phase A was 0.1% formic acid in water, and mobile phase B was 0.1% formic acid in 80% acetonitrile. A linear gradient elution program was set as follows: 0 min, 4% B; 0–2 min, 4–8% B; 2–35 min, 8–28% B; 35–55 min, 28–40% B; 55–56 min, 40–95% B; 56–66 min, 95% B. All were at a constant flow rate of 600 nL/min.

Full-MS parameters were as follows: resolution, 70,000; AGC target, 3 × 106; maximum injection time, 100 ms; scan range, *m*/*z* 100–1500. MS/MS parameters: resolution, 17,500; AGC target, 1 × 105; maximum injection time, 50 ms; top 20 precursor ions selected for fragmentation; normalized collision energy, 28 (or stepped NCE 28).

The raw mass spectrometry files were searched against the target protein database using database search software with the following parameters: fixed modification, carbamidomethyl (C); variable modifications, oxidation (M) and acetylation (peptide N-terminus); enzyme, non-specific; database, UniProt Proteome UP000283509 (*Litopenaeus vannamei*, taxon ID 6689); peptide mass tolerance, 20 ppm; fragment mass tolerance, 0.02 Da.

#### 2.10.3. BLAST Matching

The identified peptide sequences were matched with an in-house bioactive peptide sequence bank with local BLAST (Basic Local Alignment Search Tool, version 2.17.0+) using the default settings. The local peptide sequence database collected 130 antioxidant and neuroprotective peptide sequences retrieved from the literature, with these peptides originating from various organisms, including shrimps.

#### 2.10.4. pepADMET Prediction

To preliminarily assess the brain accessibility and safety of the identified bioactive peptides, peptide sequences with molecular weights below 650 Da and high homology to known antioxidant peptides (≥80% similarity) or neuroprotective peptides (≥60% similarity) were selected. Their blood–brain barrier (BBB) permeability and toxicity were predicted using the online server PepADMET (https://pepadmet.ddai.tech/). The BBB+ probability (probability of BBB penetration) and non-toxicity probability (Non-Tox) were recorded as evaluation metrics.

### 2.11. Statistical Analysis

All experiments were repeated at least three times. Data are presented as mean ± standard deviation. Differences among multiple groups were analyzed using one-way analysis of variance (ANOVA). Data analysis and graphing were performed using GraphPad Prism 9.0, Excel, and MSDIAL 4.7. The symbol “*” indicates the level of significance compared with the control group, where “*” represents *p* ≤ 0.05 (significant difference), “**” represents *p* ≤ 0.01, and “***” represents *p* ≤ 0.001.

## 3. Results

### 3.1. The Results of Three-Phase Partitioning from Pacific White Shrimp Heads

**Figure 1:**
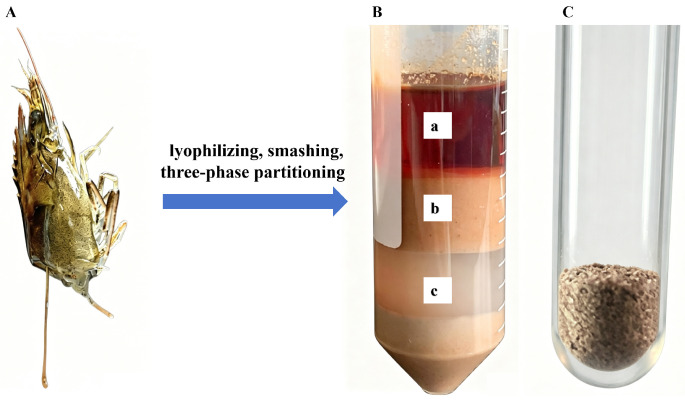
The preparation of the *Litopenaeus vannamei* shrimp head lipid and peptide. (**A**) The *Litopenaeus vannamei* shrimp head. (**B**) The three-phase partitioning separation of shrimp heads: a. lipid layer, b. protein layer, c. water-soluble layer. (**C**) Pep-SH obtained after enzymatic hydrolysis and lyophilization.

After TPP extraction, the shrimp head extract separated into three layers above the solid residue in the centrifuge tube (Figure 1B). Layer a contained nonpolar substances, including lipids, n-butanol, and pigments. Layer b consisted of a protein precipitate, while layer c contained water-soluble substances such as polysaccharides. The protein precipitate from layer b was lyophilized, enzymatically hydrolyzed, and then re-lyophilized to obtain Pep-SH (Figure 1C). The yield of PL-SH was 13.56 mg/g dry weight of shrimp head powder (2.56 g from 188.8 g). The protein precipitate (P-SH) from TPP and its enzymatic hydrolysate (Pep-SH) were obtained in adequate amounts. Since most P-SH was immediately used for enzymatic hydrolysis and exogenous enzymes were introduced during preparation, the exact yields of P-SH and Pep-SH were not determined.

### 3.2. The Antioxidant Activity of PL-SH, P-SH, and Pep-SH

A series of antioxidant activity assays were conducted on PL-SH, P-SH, and Pep-SH, including DPPH, ABTS^+^, and hydroxyl free radical scavenging assays.

**Figure 2:**
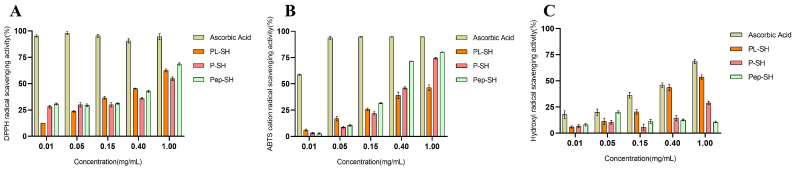
Antioxidant activities of PL-SH, P-SH, and Pep-SH. (**A**) DPPH radical scavenging activity of PL-SH, P-SH, Pep-SH, and positive control ascorbic acid. (**B**) ABTS^+^ radical cation scavenging activity of PL-SH, P-SH, Pep-SH, and positive control ascorbic acid. (**C**) Hydroxyl radical scavenging activity of PL-SH, P-SH, Pep-SH, and positive control ascorbic acid. Data are expressed as mean ± SD (*n* = 3).

The results showed that all three substances exhibited dose-dependent DPPH radical scavenging activity (Figure 2A). Pep-SH (IC_50_ = 2.72 ± 0.15 mg/mL) achieved a scavenging rate of 69% at 1 mg/mL, PL-SH (IC_50_ = 2.64 ± 0.12 mg/mL) reached 63% at 1 mg/mL, and P-SH (IC_50_ = 3.87 ± 0.26 mg/mL), the least potent among the three, attained only 56% at the same concentration.

In the ABTS^+^ radical scavenging assay, all three substances also showed concentration-dependent activity (Figure 2B). Pep-SH (IC_50_ = 2.37 ± 0.05 mg/mL) exhibited a scavenging rate of 80% at 1 mg/mL, P-SH (IC_50_ = 2.62 ± 0.04 mg/mL) achieved 75%, while PL-SH (IC_50_ = 3.17 ± 0.13 mg/mL), the weakest among the three, reached only 49% at the same concentration.

In the hydroxyl radical scavenging assay (Figure 2C), PL-SH (IC_50_ = 2.87 ± 0.11 mg/mL) achieved a scavenging rate of 55% at 1 mg/mL, P-SH reached 30%, and Pep-SH was the least effective, attaining only 11% at the same concentration.

These results demonstrate that PL-SH, Pep-SH, and P-SH all possess considerable antioxidant activity. Given that Pep-SH exhibited overall higher antioxidant activity than non-hydrolyzed P-SH, PL-SH and Pep-SH were selected for subsequent neuroprotection and anti-neuroinflammation studies.

### 3.3. The Neuroprotection of PL-SH and Pep-SH Against the Damage of Aβ_25–35_ to HT-22 Cells

**Figure 3:**
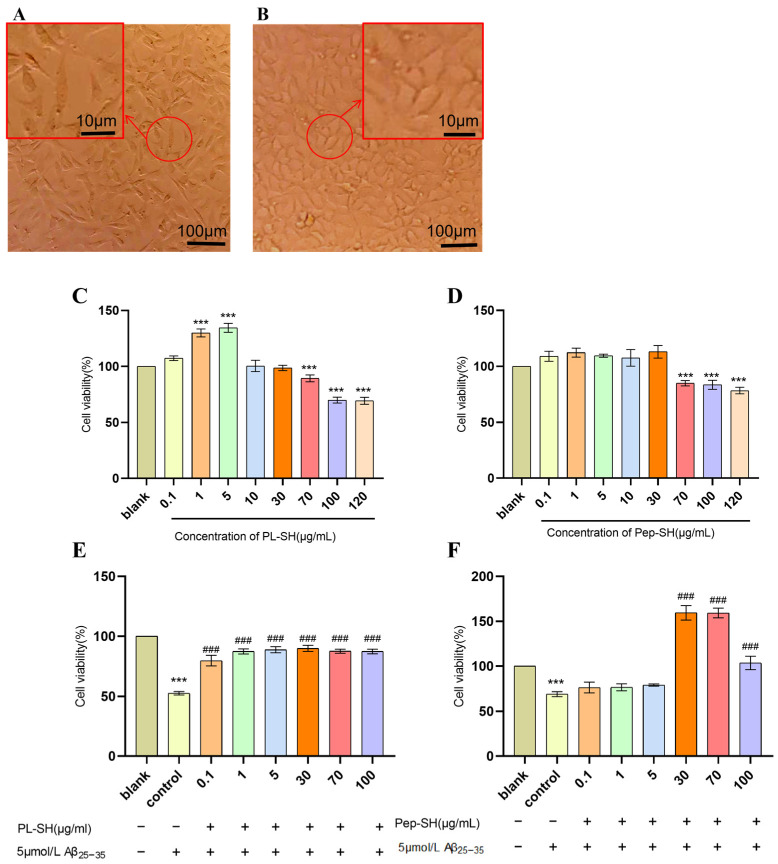
Neuroprotective activities of PL-SH and Pep-SH. (**A**) Normal morphology of HT22 cells. (**B**) Morphology of HT22 cells following 2 h Aβ_25–35_ treatment. (**C**) The cytotoxic effect of PL-SH on HT-22 cells. (**D**) The cytotoxic effect of Pep-SH on HT-22 cells. (**E**) The effect of PL-SH on the viability of HT-22 cells treated with Aβ_25–35_. (**F**) The effect of Pep-SH on the viability of HT-22 cells treated with Aβ_25–35_. Data are expressed as mean ± SD (*n* = 3). “***”, Compared with the blank group, *p* < 0.001; “###”, Compared with the control group, *p* < 0.001.

The results showed that normal HT-22 cells, which exhibited a polygonal morphology, became more rounded after Aβ_25–35_ treatment (Figure 3A,B). Both PL-SH and Pep-SH did not inhibit HT-22 cell growth at concentrations ranging from 0.1 to 30 μg/mL but significantly inhibited growth at concentrations above 70 μg/mL (Figure 3C,D).

**Figure 4:**
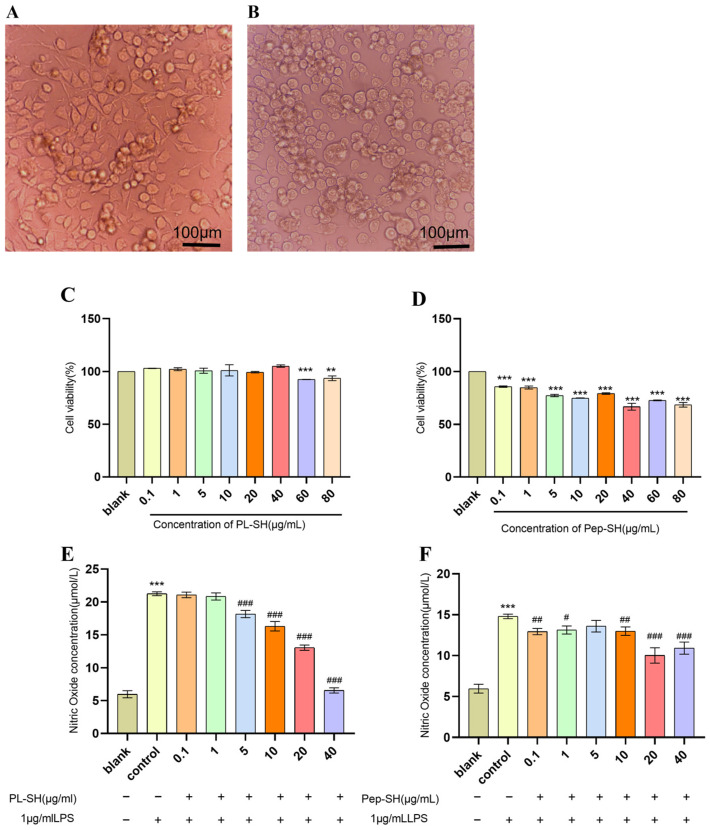
Morphological changes of BV-2 cells before and after stimulation and the anti-neuroinflammatory assay of PL-SH and Pep-SH. (**A**) Resting state of BV2 cells. (**B**) Activated state of BV2 cells. (**C**) The cytotoxic effect of PL-SH on BV-2 cells. (**D**) The cytotoxic effect of Pep-SH on BV-2 cells. (**E**) The effect of PL-SH on the production of NO in BV-2 cells treated with LPS. (**F**) The effect of Pep-SH on the production of NO in BV-2 cells treated with LPS. Data are expressed as mean ± SD (*n* = 4). “**”, Compared with the blank group, *p* < 0.01; “***”, *p* < 0.001; “#”, Compared with the control group, *p* < 0.05; “##”, *p* < 0.01; “### “, *p* < 0.001.

In this experiment, cells were first exposed to 5 μmol/L Aβ_25–35_ for 2 h to induce damage, followed by sample treatment. Compared with the blank group (untreated cells), the control group (Aβ_25–35_ only) exhibited a significant decrease in cell viability. Notably, PL-SH at concentrations ranging from 0.1 to 100 μg/mL significantly increased cell viability compared to the Aβ_25−35_-treated control (Figure 3E), suggesting a protective effect. Pep-SH at concentrations between 30 and 100 μg/mL also improved cell viability (Figure 3F). However, the moderate cytotoxicity of Pep-SH towards BV-2 cells (Figure 4D) warrants caution when interpreting its neuroprotective potential.

### 3.4. The Contribution of PL-SH and Pep-SH to the Alleviation of BV-2 Cell Activation by LPS

When not stimulated by LPS, BV-2 cells typically exhibit a ramified morphology, characterized by a small cell body with multiple slender protrusions. This morphology, referred to as “ramified”, represents the resting state of microglia (Figure 4A), whose primary function is to monitor the surrounding environment. However, upon LPS stimulation, BV-2 cells undergo significant morphological changes. The cell body becomes noticeably enlarged, while the protrusions shorten and thicken, resulting in a more rounded, amoeboid shape (Figure 4B) [28]. This transformation is a typical feature of activated microglia, indicating that the cells have entered an inflammatory state.

The results (Figure 4C,D) indicated that PL-SH had no cytotoxicity toward BV-2 cells at concentrations below 40 μg/mL but exhibited weak cytotoxicity at higher doses. Pep-SH showed moderate toxicity to BV-2 cells at concentrations ranging from 0.1 to 80 μg/mL. Therefore, a concentration range of 0.1–40 μg/mL was used to evaluate the effects of PL-SH and Pep-SH on LPS-induced NO production in BV-2 cells. The NO concentration in the blank group was extremely low, averaging 6 μmol/L. After LPS stimulation, the NO concentration in BV-2 cells increased significantly. Intriguingly, cells treated with PL-SH at concentrations of 5–40 μg/mL showed a significant dose-dependent decrease in NO concentration (Figure 4E). Although Pep-SH treatment also led to a weak reduction in NO levels at concentrations of 0.1–40 μg/mL, the NO inhibitory activity of this sample was doubtful, given its weak cytotoxicity at the same concentrations (Figure 4F). This assay revealed the anti-neuroinflammatory potential of PL-SH under the present experimental conditions.

### 3.5. The Constituents of PL-SH Revealed by TLC and LC-MS/MS Analysis

**Figure 5:**
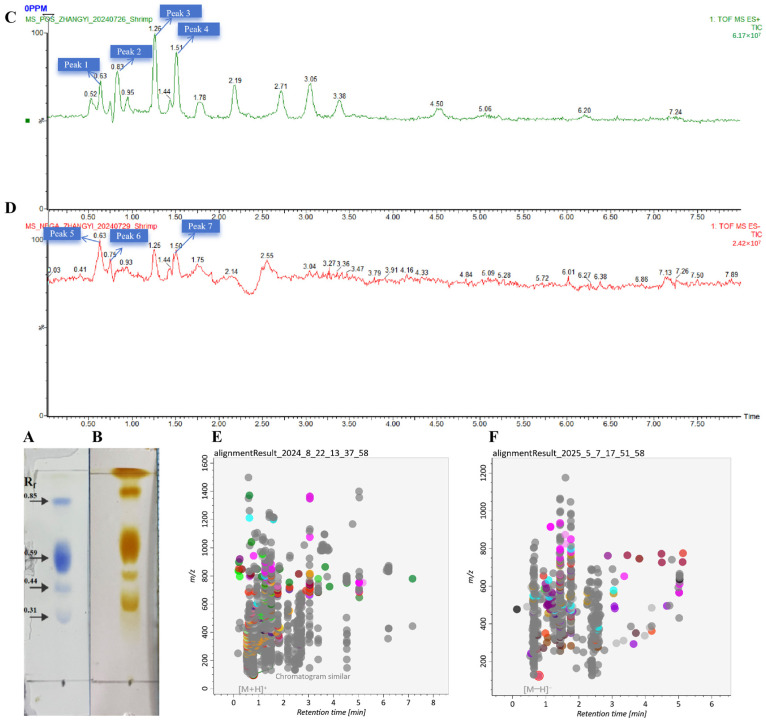
Compositional analysis of PL-SH by thin-layer chromatography (TLC) and lipidomics based on liquid chromatography-mass spectrometry (LC-MS). (**A**) TLC plate stained by phosphomolybdic acid reagent. (**B**) TLC plate stained by iodine vapor. (**C**) Total ion chromatogram of LC-MS in positive ion mode. (**D**) Total ion chromatogram of LC-MS in negative ion mode. (**E**) Lipid fingerprinting analysis by MSDIAL based on positive ion mode LC-MS data. (**F**) Lipid fingerprinting analysis by MSDIAL based on negative ion mode LC-MS data. The colors of the dots represent the lipid classes automatically annotated by the MS-DIAL software (e.g., phospholipids, sphingolipids, glycerolipids, etc.).

Phosphomolybdic acid staining is a widely used TLC method for detecting phospholipids. Its principle is that organic phosphorus in the lipid sample is released by sulfuric acid hydrolysis and then reacts with ammonium molybdate to form phosphomolybdic acid, which is subsequently reduced by L-ascorbic acid to produce characteristic molybdenum blue spots [29]. Compared with Figure 5B, which shows the total lipid components stained by iodine vapor, phosphomolybdic acid staining (Figure 5A) revealed typical phospholipid spots with Rf values of 0.31, 0.44, 0.59, and 0.85, as important constituents of PL-SH.

Furthermore, LC-MS/MS data in both positive and negative ion modes were collected for PL-SH, and lipidomics annotation was performed using MS-DIAL software. The results revealed an exceptionally diverse array of lipid species in PL-SH, with 1095 and 686 molecular species detected in positive and negative ion modes, respectively (Figure 5E,F).

The total ion chromatograms (TICs) in positive and negative ion modes (Figure 5C,D) presented several major peaks. For example, Peak 1 with a retention time (Rt) of 0.63 min contained a phytosphingosine (SPB 14:1;3O), accounting for 0.031% of the total ion abundance, along with a hexosylceramide-hydroxylated sphingosine (HexCer 18:3;2O/24:6;O), accounting for 0.003%, as well as numerous unknown substances. Peak 2 (Rt = 0.83 min) included a phytosphingosine (SPB 16:0;3O), representing 0.285% of the total ion abundance. Peak 3 (Rt = 1.257 min) contained a sphingomyelin (SM 28:5;2O) at 0.02%. Peak 4 (Rt = 1.517 min) contained a phosphatidylinositol (PI 34:6) at 0.002%. In negative ion mode, Peak 5 (Rt = 0.63 min) contained fatty acid esters of hydroxy fatty acids (AAHFAs 15:4/15:4;O), accounting for 8.3% of the total ion abundance. Peak 6 (Rt = 0.75 min) contained an oxidized fatty acid (FA 18:3;4O) at 0.021%. Peak 7 (Rt = 1.501 min) contained a fatty acid (FA 18:0) at 0.152%, along with many unknown substances. More details on the main lipid annotations are provided in Supplementary Tables S1 and S2.

**Figure 6:**
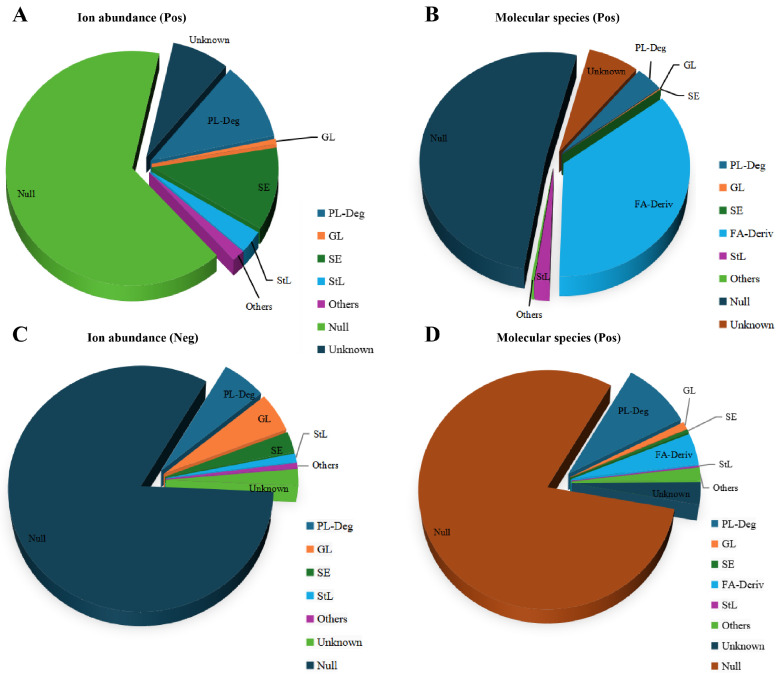
Distribution of ion abundance and molecular species of different lipid classes in PL-SH. (**A**) Distribution of ion abundance of lipid classes in positive ion mode. (**B**) Distribution of molecular species of lipid classes in positive ion mode. (**C**) Distribution of ion abundance of lipid classes in negative ion mode. (**D**) Distribution of molecular species of lipid classes in negative ion mode. PL-Deg: phospholipid degradation products; GL: glycerolipids; SE: sterol esters; StL: sterol lipids; FA-Deriv: fatty acid derivatives; Others (+mode): carnitine, dihydrosphingosine, erucamide, dodecamethylcyclohexasiloxane; Others (−mode): diferuloylputrescine, β-nicotinamide adenine dinucleotide, taurine, inosine; Unknown: mostly refers to metabolites recorded in the RIKEN database that are derived from cells and animal tissues and have specific molecular masses but whose structures are not specified; Null: unknown metabolites for which the lipidomics function of the MS-DIAL software could not establish molecular formulae and thus could not annotate.

Overall, Figure 6 shows the distribution of different lipid/metabolite classes in terms of ion abundance (Figure 6A,C) and molecular species numbers (Figure 6B,D). The metabolites annotated in positive ion mode were mainly derived from phospholipids and their degradation products, sphingolipids, and glycerolipids, with a few from sterol esters and other miscellaneous lipids. In contrast, the metabolites annotated in the negative ion mode came from a broader range of categories, primarily fatty acids and their derivatives, phospholipids and their degradation products, and sterol lipids, along with a few from sphingolipids, glycosylglycerides, and other miscellaneous lipids. Notably, a large proportion of metabolites remained annotated as unknown structures or null (i.e., no annotation) in both ion modes.

Detailed category statistics (Table 1 and Table 2) revealed diverse subcategories for different lipid classes.

For instance, phospholipids identified in both positive and negative ion modes included various glycerophospholipids, lysophospholipids, sphingophospholipids, ether glycerophospholipids, and ether lysophospholipids. In terms of molecular species numbers, phospholipids accounted for 5.39% (59 species) of the total in positive ion mode and 9.04% (62 species) in negative ion mode. In terms of ion abundance, they accounted for 10.74% of the total in positive ion mode and 3.62% in negative ion mode. At the subcategory level, sphingomyelin (SM) dominated the lipid profile (7.67%, +mode), and multiple bioactive minor constituents collectively contributed 3.68% to the total ion abundance: phosphatidylinositol (PI, 1.13%, +mode), lysophosphatidylglycerol (LysoPG, 0.79%, −mode), lysophosphatidylcholine (LysoPC, 0.78%, +mode), lysophosphatidylethanolamine (LysoPE, 0.65%, −mode), hydroxyphosphatidylethanolamine (OxPE, 0.58%, −mode), ether lysophosphatidylglycerol (EtherLysoPG, 0.46%, −mode), and hydroxyphosphatidylinositol (OxPI, 0.41%, −mode).

As for the non-phospholipid sphingolipids, which include cerebrosides, ceramides, and sphingosines, their total molecular species numbers accounted for 4.93% (54 species, +mode) and 1.02% (7 species, −mode), respectively. Their total ion abundance proportions were 11.41% (+mode) and 0.04% (−mode), respectively. Among them, the subcategories with higher ion abundance included dihydrosphingosine (DHSph, 8.23%, +mode), phytosphingosine (PhytoSph, 2.09%, +mode), HexCer-AP (0.59%, +mode), and N-acyl-sphingosine ceramide (Cer-NS, 0.17%, +mode).

In addition, the following lipid classes exhibited relatively high ion abundance: saccharolipids (SL, 2.91%, +mode), diacylglycerols (DG, 0.29%, +mode), VAE (0.20%, +mode), hydroxy fatty acid esters (FAHFA, 35.06%, −mode), sterol hexosides (SHex, 1.75%, −mode), and fatty acids (FA, 0.66%, −mode).

The analysis based on TLC and lipidomics suggests that, aside from unidentified substances, phospholipids, sphingolipids, fatty acids, and sterol esters are likely the major components of PL-SH.

### 3.6. The Molecular Weight Determination of Pep-SH

**Figure 7:**
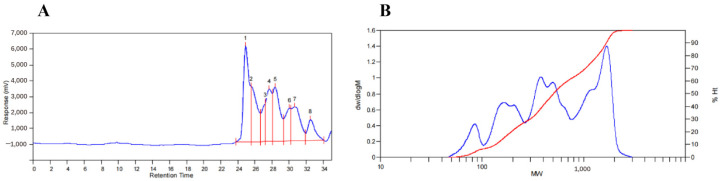
The molecular weight distribution diagram of Pep-SH determined by gel permeation chromatography (GPC). (**A**) Eluent profile graph of GPC: the horizontal coordinate represents time, and the vertical coordinate represents signal intensity, with a total of 8 peaks detected. (**B**) Molecular weight distribution curve and cumulative curve: the horizontal coordinate represents the molecular weight, and the vertical coordinate is divided into two sides. The left side represents the signal intensity (corresponding to the blue peak curve) of each molecular weight, while the right side represents the cumulative result (corresponding to the red curve).

In the gel permeation chromatography (GPC) elution profile (Figure 7A), a total of eight peaks (peaks 1–8) with progressively decreasing molecular weights were detected in Pep-SH. In the molecular weight distribution curve (Figure 7B), the molecular weight distributions of these peaks, ranging from 50 Da to 3000 Da, are presented in reverse order along with the cumulative integral curve to indicate the proportional composition.

Furthermore, different statistical methods revealed more accurate molecular weight information, including Mp (peak molecular weight), Mn (number-average molecular weight), Mw (weight-average molecular weight), Mz (z-average molecular weight), Mz + 1 (the next higher molecular weight after Mz), Mv (viscosity-average molecular weight), and PD (polydispersity index, PD = Mw/Mn) [30] (Table 3), which collectively characterized the overall properties of Pep-SH. In particular, the PD value (Mw/Mn) indicates the uniformity of the peptides, which is closely related to the reproducibility of their bioactivity and physicochemical properties. Mp indicates the dominant peptide size in each peak. Mn reflects the contribution of small peptides, while Mw is weighted toward larger ones. Mz and Mz + 1 are sensitive to trace amounts of high-molecular-weight species. Mv relates to the solution behavior (e.g., diffusion, viscosity). Together with PD, these parameters fully characterize the molecular weight distribution, which underpins batch consistency and functional reproducibility.

In summary, the molecular weight range of peptides in Pep-SH spanned from 80 to 1600 Da, indicating that the shrimp head proteins had been largely hydrolyzed into oligopeptides. The PD values (all close to 1) indicated the molecular weight homogeneity of the peptides within each peak. Peak 1 exhibited the highest abundance in Pep-SH, with an approximate molecular weight of 1600 Da.

### 3.7. LC-MS/MS Identification of Peptides in Pep-SH

**Figure 8:**
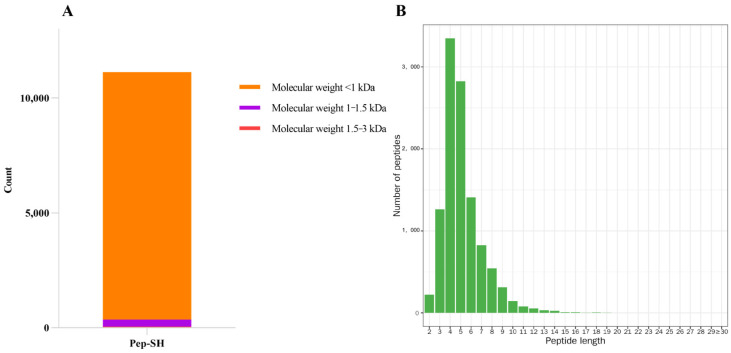
The molecular weight (**A**) and peptide length (**B**) distribution of Pep-SH determined by LC-MS/MS-based sequencing.

The raw mass spectrometry files were processed using database search software to obtain peptide identification results. Consistent with the GPC results, most of the peptides fell within a molecular weight range of less than 1 kDa and a length range of 3–9 amino acid residues (Figure 8). The top ten peptides ranked by identification confidence are detailed in Table 4.

To retrieve bioactive peptide sequences from Pep-SH, all peptide sequences identified by database searching were compared against reported bioactive peptide sequences using a local BLAST algorithm. A total of 93 unique matches with sequence identity greater than 50% were identified. These matches were categorized into two groups based on their homology to known antioxidant peptides (68 matches) and neuroprotective peptides (48 matches). Table 5 and Table 6 lists some of the sequences with higher similarity, and detailed information is provided in Supplementary Tables S3 and S4.

Twelve peptides showed 100% sequence similarity to known bioactive peptides in the database. Among these matches, several were derived from *L. vannamei* proteins such as A0A423SEE9, A0A3R7PEZ1, and A0A423T7C2. These high-similarity matches provide strong evidence for the presence of known bioactive peptide motifs in Pep-SH. Additionally, a large number of other peptides were identified with high sequence similarity (≥80%), indicating the existence of structural analogues or variants of known bioactive peptides. For instance, a peptide derived from A0A423SBQ1 (mass = 895.4763 Da) exhibited 83.33% similarity to a known neuroprotective peptide (SLPSLPEPV).

To preliminarily evaluate the brain accessibility and safety of the identified bioactive peptides, we performed in silico prediction of BBB permeability and toxicity. Only those peptides with molecular weight below 650 Da, along with high sequence similarity to known antioxidant (≥80%) or neuroprotective (≥60%) peptides, were selected for prediction (https://pepadmet.ddai.tech/ (accessed on 26 May 2026)) (Table 7 and Table 8). This threshold was chosen because low molecular weight generally favors passive BBB penetration, and high similarity suggests potential bioactivity. Among the predicted peptides, IIAPPE (antioxidant) showed acceptable BBB permeability and low toxicity. For neuroprotective activity, SLPSVP, SPVSLP, SIPVSP, and AGPGVSP exhibited the most favorable combination of BBB+ probability (>0.54) and high non-toxicity probability (>0.93), making them the best candidates for further study.

The identification of peptides with high homology to known antioxidant and neuroprotective peptides directly supports the strong antioxidant (Section 3.1) and neuroprotective (Section 3.2) activities observed in the Pep-SH fraction. The presence of these putative bioactive peptides indicates that the bioactivities of Pep-SH are likely mediated by a combination of these identified compounds. In conclusion, the LC-MS/MS analysis and subsequent database search successfully identified a range of peptides in Pep-SH with significant homology to known antioxidant and neuroprotective peptides. These results provide a molecular basis for the potent bioactivities observed in this study and highlight the potential of shrimp head-derived peptides as a source of functional ingredients for brain health.

## 4. Discussion

Currently, Alzheimer’s disease (AD) and age-related cognitive decline have become severe global health challenges, driving the search for novel functional ingredients that can prevent or ameliorate neuronal damage and neuroinflammation [38]. Since its introduction to China in 1988, *Litopenaeus vannamei*, known for its high economic value and adaptability, has become China’s leading mariculture shrimp species and one of the most significant species globally [39]. Its processing generates substantial amounts of head by-products, which are rich in proteins and lipids [40]. Previous studies have primarily focused on the antioxidative [41], immunomodulatory [42], cardiovascular [43], and antitumor activities [44] of shrimp-head-derived components, but investigations on their potential neuroprotective and anti-neuroinflammatory effects remain limited.

In this study, phospholipids (PL-SH) and peptides (Pep-SH) were successfully extracted from shrimp heads using three-phase partitioning followed by proteolysis. Both PL-SH and Pep-SH exhibited strong antioxidant activities in DPPH, ABTS^+^, and hydroxyl radical scavenging assays, indicating their potential to counteract oxidative stress. Given that oxidative stress is a key pathological factor in neurodegenerative diseases, this property may partly explain the biological effects of PL-SH and Pep-SH observed in cellular models.

Moreover, their protective effects against Aβ-induced cytotoxicity in HT-22 cells highlight their potential to mitigate amyloid-related neurotoxicity—a central event in AD pathogenesis. In particular, the anti-neuroinflammatory effects of PL-SH are noteworthy, as chronic neuroinflammation driven by activated microglia is a hallmark of AD and other neurodegenerative disorders [45]. The inhibitory effect of PL-SH on LPS-induced NO production in BV-2 cells suggests its potential to modulate microglial activation and attenuate neuroinflammatory cascades, although this observation is based solely on NO measurement and requires confirmation using additional inflammatory markers (e.g., TNF-α, IL-6, iNOS).

Chemical characterization of PL-SH via TLC staining and LC-MS/MS-based lipidomics revealed a complex lipid profile, including phospholipids, sphingolipids, glycerolipids, sterol esters, fatty acids, and unknown components. Among the annotated metabolites, sphingomyelin (SM), phosphatidylinositol (PI), lysophosphatidylglycerol (LPG), and lysophosphatidylcholine (LPC) were identified as major components. Lipids such as glycerophospholipids, sphingomyelin, sphingolipids, and cholesterol constitute the structural lipids of synapses and play pivotal roles in regulating signal transduction, synaptogenesis, neurogenesis, neurite outgrowth, and long-term potentiation (LTP), all of which are essential for neural regeneration [46,47]. Specifically, PI serves as a key precursor for the phosphoinositide signaling involved in neuroinflammation and neuronal survival [48], while SM is essential for myelin stability and synaptic plasticity [49]. In negative-ion mode, PL-SH was found to contain a large repertoire of fatty acids (FAs), the most abundant organic compounds in the brain, accounting for ~60% of its dry weight, of which roughly 20% are polyunsaturated fatty acids (PUFAs) [50]. The dominant PUFAs in brain tissue are omega-3 docosahexaenoic acid (DHA) and omega-6 arachidonic acid [51]. Omega-3 PUFAs, primarily DHA and eicosapentaenoic acid, exhibit strong anti-inflammatory and inflammation-resolving properties [52], providing compelling evidence for the anti-neuroinflammatory efficacy of PL-SH. Notably, FAHFAs (fatty acid esters of hydroxy fatty acids) accounted for 35.06% of the total ion abundance in PL-SH (Table 2). FAHFAs are endogenous lipokines with documented anti-inflammatory and insulin-sensitizing properties. Emerging evidence also suggests they exert antioxidant and neuroprotective effects, partly by modulating immune responses and promoting autophagy [53]. Thus, the abundant FAHFAs in PL-SH may contribute to its anti-neuroinflammatory and neuroprotective activity. Marine-derived lipids from krill, fish, and sea cucumber have also been reported to possess neuroprotective and anti-inflammatory properties [54,55]. For instance, krill oil, rich in EPA/DHA-enriched phospholipids, attenuates neuroinflammation and improves cognitive deficits in AD-like models [54]. Similarly, sea cucumber EPA-enriched phospholipids protect against Aβ-induced neurotoxicity via antioxidant and anti-apoptotic pathways [55]. The present study extends these findings by identifying FAHFAs (accounting for 35.06% of total ion abundance) as a major component in shrimp head lipids, which has not been previously emphasized in marine lipid research. Moreover, our TPP-based extraction provides a green, one-step approach to obtain shrimp head lipids without organic-solvent intensive procedures. Regarding safety, the representative lipids identified in PL-SH—including sphingomyelin, phosphatidylinositol, and omega-3 PUFAs—are natural membrane constituents and have a long history of safe use as dietary supplements [56]. Although systematic toxicological data for FAHFAs are lacking, their endogenous presence in common foods (e.g., olive oil, milk) suggests a low safety concern [57].

Similarly, GPC, LC-MS/MS, and database matching analysis revealed that Pep-SH contains a variety of low-molecular-weight peptides (80–1600 Da), especially short oligopeptides. Smaller peptides, particularly those within the 0.5–3 kDa range, are considered key factors influencing the antioxidant activity of protein hydrolysates [58], which provides a significant explanation for the strong antioxidant and neuroprotective activities of Pep-SH. Notably, some Pep-SH peptide sequences showed high homology to known antioxidant and neuroprotective peptides. The identification of these peptides with 80–100% similarity to established bioactive sequences also provides a molecular basis for the biological activities observed in earlier experiments. The low molecular weight characteristic, together with their similarity to known bioactive peptides, further supports the potential of Pep-SH as a functional ingredient for promoting brain health [59]. To preliminarily assess their brain accessibility and safety, we performed in silico prediction of BBB permeability and toxicity for selected low-molecular-weight peptides (<650 Da) with high homology to known bioactive peptides (Table 6 and Table 7). Most predicted peptides were BBB+ (62.5% of antioxidant and 85.7% of neuroprotective peptides), with low predicted toxicity. Notably, four neuroprotective peptides (SLPSVP, SPVSLP, SIPVSP, AGPGVSP) exhibited favorable BBB+ probabilities (0.55–0.61) and high non-toxicity probabilities (>0.93), supporting their potential for further in vivo study. Regarding allergenicity, no direct experimental data are available; however, the majority of these peptides originate from endogenous shrimp proteins, suggesting a potentially low risk. Nevertheless, we acknowledge that this is only a theoretical inference. Comprehensive allergenicity assessment—including in silico prediction and in vitro/in vivo testing—is required before any human consumption of PL-SH or Pep-SH can be considered.

Compared with other marine-derived bioactive components, especially those from wild animals or plants, shrimp head-derived PL-SH and Pep-SH offer a sustainable and cost-effective source of neuroprotective ingredients. The use of three-phase partitioning (TPP) for the simultaneous extraction of lipids and proteins also aligns with green processing principles [60]. Compared with methods for extracting phospholipids from shrimp heads using ethanol, n-hexane, and acetone solutions [61], as well as classical organic solvent extraction techniques such as the Folch method, Bligh and Dyer method, and methyl tert-butyl ether (MTBE) method [62], our process is more convenient and significantly enhances the value of shrimp processing waste. The remaining polysaccharides (chitin) derived from TPP can also serve as industrial chemical materials with broad applications.

It should be noted that the lipidomic analysis identified a large proportion of metabolites as “unknown” or “null” (Table 1 and Table 2), indicating that many lipid species remain to be structurally elucidated. Moreover, no absolute quantification was performed; therefore, the relative ion abundance of each lipid class may not precisely reflect its actual concentration in PL-SH. Regarding peptide identification, the bioactive peptide sequences were assigned by LC-MS/MS and BLAST homology. Their actual neuroprotective activities have not yet been confirmed by chemically synthesized peptides. Future studies should focus on purifying and characterizing the individual active components.

Nevertheless, this study has certain limitations. First, the anti-inflammatory evaluation relied solely on nitric oxide (NO) measurement; other key inflammatory mediators such as iNOS, COX-2, TNF-α, and IL-6 were not examined. Similarly, the neuroprotective assessment was based only on cell viability (MTT assay), without measuring reactive oxygen species (ROS) or apoptotic markers (e.g., caspase-3, Bax/Bcl-2). Moreover, Pep-SH exhibited moderate cytotoxicity towards BV-2 cells at concentrations above 40 μg/mL (Figure 4D), which should be considered when interpreting its neuroprotective effects. Therefore, the observed activities should be regarded as preliminary, and more detailed mechanistic and safety studies are required. Second, the molecular mechanisms underlying the observed neuroprotective and anti-inflammatory effects remain largely unknown. We did not investigate interactions with receptors or ion channels, nor did we analyze intracellular signaling cascades (e.g., MAPK, NF-κB, PI3K/Akt) or apoptotic/autophagic processes. Third, no in vivo studies were conducted. The current findings are based exclusively on in vitro cell culture experiments, and their relevance to living organisms—especially in the context of Alzheimer’s disease—requires confirmation using animal models. Transgenic mouse lines such as APP/PS1, 5×FAD, and Tg2576, which recapitulate key pathological features of AD (Aβ accumulation, tau pathology, neurodegeneration), should be employed in future research. Fourth, since only the yield of lipid PL-SH has been calculated for this comprehensive extraction process, the protein P-SH and its enzymolytic product Pep-SH should also be quantified in future study. Fifth, the stability of PL-SH and Pep-SH during storage, in functional food matrices, and their resistance to proteolytic digestion have not been evaluated. These are essential for any practical application. All the above limitations point to clear directions for future work, including comprehensive mechanism dissection, in vivo efficacy studies, and rigorous safety and stability assessments.

## 5. Conclusions

In summary, phospholipids (PL-SH) and peptides (Pep-SH) extracted from the head of *Litopenaeus vannamei* using three-phase partitioning followed by proteolysis exhibit antioxidant activities and show potential neuroprotective and anti-neuroinflammatory effects in vitro. These preliminary effects require further mechanistic and safety studies. Among the identified constituents, several lipids and peptides are particularly noteworthy for their documented beneficial properties. In PL-SH, sphingomyelin (SM), phosphatidylinositol (PI), fatty acids (FAs), and FAHFAs are recognized for their neuroprotective and anti-inflammatory properties. In Pep-SH, the BBB+ and low-toxic peptide GDDAPR shows 100% sequence similarity to a known antioxidant peptide, while the BBB+ and low-toxic peptide GPGLGP shares 83% similarity with a reported neuroprotective peptide, suggesting their potential contributions to the observed neuroprotective and anti-inflammatory activities. These findings underscore the potential of shrimp head by-products as a valuable source of functional ingredients for brain health. However, all conclusions are based on in vitro experiments only. Further in vivo validation using AD animal models, detailed mechanistic studies (including signaling pathways and receptor interactions), assessments of bioavailability and blood–brain barrier permeability, as well as stability and safety evaluations, are strictly required before any application can be claimed. Future research should address these aspects to translate the current findings into practical nutraceutical or functional food products.

## Figures and Tables

**Table 1:** The lipidomics annotation results of PL-SH based on positive ion mode mass spectrometry data.

Lipid Composition	Molecular Species Number	Relative Abun- Dance to Total Ion Current/%	Lipid Composition	Molecular Species Number	Relative Abun- Dance to Total Ion Current/%
Phospholipids and Their Degradation Products			Sphingolipids		
EtherPE	3	0.0738	Cer-NS	4	0.1700
EtherLysoPE	1	0.0182	Subtotal	54	11.4098
LysoPC	6	0.7833	Glycerolipids		
LysoPE	2	0.4589	DG	7	0.2924
PC	2	0.0250	MG	9	0.1895
PE	1	0.0048	OxTG	5	0.0575
PI	17	1.1292	TG	6	0.1576
PS	5	0.2735	TG-EST	2	0.0176
SM	12	7.6655	Subtotal	29	0.7146
NAE	5	0.1320	Sterolester		
NAGly	3	0.0322	ST	1	0.0108
NAGlySer	2	0.1422	SL	11	3.0282
Subtotal	59	10.7386	Subtotal	12	3.0389
Sphingolipids			Others		
HexCer-AP	9	0.5919	VAE	2	0.2045
HexCer-HS	4	0.1202	Others	5	1.4175
HexCer-NS	1	0.0220	Subtotal	7	1.6220
DHSph	12	8.2270	Unknown and Null		
PhytoSph	19	2.0891	Unknown	23	7.4056
Cer-HDS	1	0.0615	Null	911	65.0705
Cer-HS	1	0.0393	Subtotal	934	72.4761
Cer-NDS	3	0.0888	Total	1095	100

Note: EtherPE: ether phosphatidylethanolamine; EtherLysoPE: ether lysophosphatidylethanolamine; LysoPC: lysophosphatidylcholine; LysoPE: lysophosphatidylethanolamine; PC: phosphatidylcholine; PE: phosphatidylethanolamine; PI: phosphatidylinositol; PS: phosphatidylserine; SM: sphingomyelin; NAE: n-acylethanolamine; NAGly: n-acylglycine; NAGlySer: n-acylglycine serine; HexCer-AP: hexosylceramide-aminoalcohol phosphate; HexCer-HS: hexosylceramide-hydroxylated sphingosine; HexCer-NS: hexosylceramide-non-hydroxylated sphingosine; DHSph: dihydrosphingosine; PhytoSph: phytosphingosine; Cer-HDS: ceramide-dihydrosphingosine; Cer-HS: ceramide-hydroxylated sphingosine; Cer-NS: ceramide-non-hydroxylated sphingosine; Cer-NDS: ceramide-non-dihydrosphingosine; DG: diacylglycerol; MG: monoacylglycerol; OxTG: oxidized triacylglycerol; TG: triacylglycerol; TG-EST: triacylglycerol ester; ST: sterol; SL: sulfatide; VAE: very long-chain acyl-coa ethanolamide; Others: carnitine, dihydrosphingosine, erucamide, dodecamethylcyclohexasiloxane; Unknown: mostly refers to metabolites recorded in the RIKEN database that are derived from cells and animal tissues and have specific molecular masses but whose structures are not specified; Null: unknown metabolites for which the lipidomics function of the MS-DIAL software could not establish molecular formulae and thus could not annotate.

**Table 2:** The lipidomics annotation results of PL-SH based on negative ion mode mass spectrometry data.

Lipid Composition	Molecular Species Number	Relative Abun- Dance to Total Ion Current/%	Lipid Composition	Molecular Species Number	Relative Abun- Dance to Total Ion Current/%
Phospholipids and Their Degradation Products			Sphingolipids		
EtherPE	1	0.0197	HexCer-HS	1	0.0007
EtherPG	1	0.0036	Subtotal	7	0.0368
LysoPC	1	0.0272	Sterolester		
PA	1	0.0095	SHex	1	1.7463
LysoPE	7	0.6475	SSulfate	1	0.0445
LysoPI	3	0.0797	DCAE	1	0.0051
NAE	3	0.1288	Subtotal	3	1.7959
NAGlySer	5	0.0450	Fatty Acids and Their Derivatives		
OxPE	5	0.5799	FA	7	0.6591
OxPG	4	0.0855	OxFA	9	0.1810
OxPI	8	0.4083	FAHFA	13	35.0550
OxPS	2	0.0170	Subtotal	29	35.8951
PG	3	0.0583	Glycerolipids		
PI	2	0.0111	EtherSMGDG	1	0.1379
PS	3	0.1346	Subtotal	1	0.1379
EtherLPG	1	0.4602	Others		
LysoPG	6	0.7858	Others	12	0.3429
LysoPS	6	0.1155	Subtotal	12	0.3429
Subtotal	62	3.6171	Unknown and Null		
Sphingolipids			Unknown	21	6.8023
HexCer-NS	3	0.0247	Null	551	51.3720
HexCer-NDS	1	0.0070	Subtotal	572	58.1742
HexCer-AP	1	0.0041	Total	686	100
HexCer-HDS	1	0.0003			

Note: EtherPE: ether phosphatidylethanolamine; EtherPG: ether phosphatidylglycerol; LysoPC: lysophosphatidylcholine; PA: phosphatidic acid; LysoPE: lysophosphatidylethanolamine; LysoPI: lysophosphatidylinositol; NAE: n-acylethanolamine; NAGlySer: n-acylglycylserine; OxPE: oxidized phosphatidylethanolamine; OxPG: oxidized phosphatidylglycerol; OxPI: oxidized phosphatidylinositol; OxPS: oxidized phosphatidylserine; PG: phosphatidylglycerol; PI: phosphatidylinositol; PS: phosphatidylserine; EtherLPG: ether lysophosphatidylglycerol; LysoPG: lysophosphatidylglycerol; LysoPS: lysophosphatidylserine; HexCer-NS: hexosylceramide-non-hydroxylated sphingosine; HexCer-NDS: hexosylceramide-non-dihydrosphingosine; HexCer-AP: hexosylceramide-aminoalcohol phosphate; HexCer-HDS: hexosylceramide-dihydrosphingosine; HexCer-HS: hexosylceramide-hydroxylated sphingosine; SHex: sulfatide hexoside; SSulfate: sterol sulfate; DCAE: diacylglycerol acyl ester; FA: fatty acid; OxFA: oxidized fatty acid; FAHFA: fatty acid esters of hydroxy fatty acids; EtherSMGDG: ether sphingomyelin glycosyldiacylglycerol; Others: diferuloylputrescine, β-nicotinamide adenine dinucleotide, taurine, inosine; Unknown: mostly refers to metabolites recorded in the RIKEN database that are derived from cells and animal tissues and have specific molecular masses but whose structures are not specified; Null: unknown metabolites for which the lipidomics function of the MS-DIAL software could not establish molecular formulae and thus could not annotate.

**Table 3:** Molecular weight distribution of peptides in Pep-SH determined by gel permeation chromatography (GPC).

Peak No.	Mp	Mn	Mw	Mz	Mz + 1	Mv	PD
1	1682	1572	1606	1643	1682	1601	1.0216
2	1223	974	992	1010	1028	990	1.0185
3	590	660	663	667	670	663	1.0046
4	499	494	498	503	507	498	1.0081
5	380	339	346	352	357	345	1.0207
6	206	219	221	222	224	221	1.0091
7	165	149	152	156	159	152	1.0201
8	84	78	80	82	83	79	1.0256

**Table 4:** Detailed information on the top ten peptides ranked by confidence identified through database comparison.

No.	Sequence ID	Peptide	Protein Accession	−10 lgP	Mass	Length	Area	ppm	*m*/*z*	z	RT
1	1_A0A423SXN9_PENVA_Mass=_2050.9629_Length=_18	KVTVPIVSDDEC(+57.02)RDAYGQ	A0A423SXN9|A0A423SXN9_PENVA	56.27	2050.9629	18	280,000	1.1	684.66235	3	35.8826
2	2_A0A423T5T3_PENVA_Mass=_2128.1415_Length=_20	GEKPPSIKPEEPIEGPVTKP	A0A423T5T3|A0A423T5T3_PENVA	53.22	2128.1415	20	1,150,000	1.7	710.38898	3	32.1055
3	3_A0A423SXN9_PENVA_Mass=_1543.7882_Length=_15	IVGGTDAKPGELPYQ	A0A423SXN9|A0A423SXN9_PENVA	51.48	1543.7882	15	3,130,000	0.8	772.90198	2	34.8159
4	4_A0A423SXN9_PENVA_Mass=_1763.6879_Length=_15	GHC(+57.02)VQGEDM(+15.99)NNPDYL	A0A423SXN9|A0A423SXN9_PENVA	50.58	1763.6879	15	314,000	1.6	882.85266	2	27.8836
5	5_A0A3R7Q123_PENVA_Mass=_1226.5792_Length=_11	GSHGKYPDNRP	A0A3R7Q123|A0A3R7Q123_PENVA	48.57	1226.5792	11	1,570,000	3.2	614.29883	2	9.6438
6	6_A0A423SXN9_PENVA_Mass=_1922.868_Length=_17	VTVPIVSDDEC(+57.02)RDAYGQ	A0A423SXN9|A0A423SXN9_PENVA	47.61	1922.868	17	122,000	0.3	962.44153	2	41.4958
7	7_A0A423T7C2_PENVA_Mass=_1031.3978_Length=_10	VC(+57.02)DSGDGVSH	A0A423T7C2|A0A423T7C2_PENVA	47.13	1031.3978	10	2,080,000	0.7	1032.40576	1	10.1292
8	8_A0A3R7SND6_PENVA_Mass=_1617.7742_Length=_14	DVC(+57.02)LEDTVC(+57.02)KPIVA	A0A3R7SND6|A0A3R7SND6_PENVA	46.76	1617.7742	14	173,000	0.9	809.89508	2	47.4023
9	9_X2KWE4_PENVA_Mass=_1149.603_Length=_11	SVTVPDVPSIH	X2KWE4|X2KWE4_PENVA	45.15	1149.603	11	142,000,000	1	1150.61133	1	42.5238
10	10_A0A423SXN9_PENVA_Mass=_1252.5659_Length=_12	GYGC(+57.02)ARPGYPGV	A0A423SXN9|A0A423SXN9_PENVA	44.98	1252.5659	12	8,710,000	1.2	627.29095	2	28.8183

Note: Sequence ID: unique identifier for each peptide or spectrum identified from the raw mass spectrometry data; Peptide: amino acid sequences of the identified peptides; (+57.02): cysteine carbamidomethylation, a fixed modification resulting from alkylation with iodoacetamide, which adds 57.02 Da to the residue; (+15.99): methionine oxidation, a common variable modification during sample processing, which adds 15.99 Da to the residue; Protein Accession: protein identification number of the peptide identified by database comparison; −10 lgP: significance score of peptides identified through database search; Mass: peptide molecular weight; Length: number of amino acids in the peptide; Area: peptide peak area; ppm: error between detected molecular weight and theoretical molecular weight; *m*/*z*: actually detected mass-to-charge ratio (*m*/*z*) of the peptide; z: charge state of the peptide; RT: retention time of the peptide (unit: min).

**Table 5:** Representative antioxidant peptides identified in Pep-SH by local BLAST search.

Sequence ID	−10 lgP	Peptide	Identified Bioactive Peptide Sequence	References	Similarity (%)	Length	E-Value	Bit Score	Bioactivity
50_A0A423SEE9_PENVA_Mass=_786.3144_Length=_8	36.52	DSGDGVTH	DSGDGVTHTVPIYEG	[31]	100	8	0.000301	19.6	Antioxidant
10574_A0A423T7C2_PENVA_Mass=_922.56_Length=_8	5.62	IIAPPERK	KIKIIAPPERKYSVW	[31]	100	8	0.000351	19.2	Antioxidant
1149_A0A3R7PEZ1_PENVA_Mass=_777.3908_Length=_7	20.41	TVPIYEG	DSGDGVTHTVPIYEG	[31]	100	7	0.000776	18.1	Antioxidant
190_A0A423T7C2_PENVA_Mass=_700.314_Length=_7	26.72	AGDDAPR	AGDDAPRAVF	[31]	100	7	0.001000	17.7	Antioxidant
675_A0A423T7C2_PENVA_Mass=_904.4039_Length=_9	22.15	GFAGDDAPR	AGDDAPRAVF	[31]	100	7	0.001000	17.7	Antioxidant
2962_A0A423T7C2_PENVA_Mass=_847.3824_Length=_8	16.58	FAGDDAPR	AGDDAPRAVF	[31]	100	7	0.001000	17.7	Antioxidant
6322_A0A423SEE9_PENVA_Mass=_671.2874_Length=_7	11.43	SGDGVTH	DSGDGVTHTVPIYEG	[31]	100	7	0.001000	17.7	Antioxidant
161_A0A3R7PEZ1_PENVA_Mass=_720.3694_Length=_6	27.57	TVPIYE	DSGDGVTHTVPIYEG	[31]	100	6	0.003000	16.2	Antioxidant
1306_A0A423T7C2_PENVA_Mass=_629.2769_Length=_6	19.95	GDDAPR	AGDDAPRAVF	[31]	100	6	0.003000	16.5	Antioxidant
356_A0A423SEE9_PENVA_Mass=_584.2554_Length=_6	24.05	GDGVTH	DSGDGVTHTVPIYEG	[31]	100	6	0.004000	16.2	Antioxidant
10819_A0A423TWP3_PENVA_Mass=_949.4981_Length=_9	5.36	TGGLARYVN	PGGVGGLARYT	[32]	100	6	0.006000	15.8	Antioxidant
2334_A0A423T7C2_PENVA_Mass=_638.3639_Length=_6	17.67	IIAPPE	KIKIIAPPERKYSVW	[31]	100	6	0.007000	15.4	Antioxidant
7_A0A423T7C2_PENVA_Mass=_1031.3978_Length=_10	47.13	VCDSGDGVSH	DSGDGVTHTVPIYEG	[32]	87.5	8	0.0008	18.5	Antioxidant
162_A0A3R7SUV3_PENVA_Mass=_720.3694_Length=_6	27.57	TVPLYE	DSGDGVTHTVPIYEG	[32]	83.333	6	0.006	15.4	Antioxidant
3120_A0A423T7C2_PENVA_Mass=_638.2547_Length=_6	16.3	DAYVGD	VGMGQKDSYVGDEAQSKRGILT	[32]	83.333	6	0.012000	15	Antioxidant
888_A0A423T7C2_PENVA_Mass=_570.2398_Length=_6	21.23	GDGVSH	DSGDGVTHTVPIYEG	[32]	83.333	6	0.014000	15	Antioxidant
2588_A0A3R7PTG0_PENVA_Mass=_774.3508_Length=_7	17.24	DRDGVVD	AVDINRDGVVSE	[32]	83.333	6	0.085000	13.9	Antioxidant
5216_A0A3R7MI39_PENVA_Mass=_472.2645_Length=_6	13.03	GGLGGL	PGGVGGLARYT	[31]	83.333	6	0.480000	12.7	Antioxidant
7311_A0A3R7PG97_PENVA_Mass=_626.3024_Length=_8	9.97	GPGSVGGP	PGGVGGLARYT	[31]	83.333	6	0.510000	12.7	Antioxidant
8819_A0A3R7MTS3_PENVA_Mass=_653.3384_Length=_6	7.81	TPDPKP	DPALATEPDPMPF	[31]	80	5	0.890000	11.9	Antioxidant
3440_A0A3R7LZ42_PENVA_Mass=_464.2019_Length=_6	15.75	GGFGGA	PGGVGGLARYT	[32]	80	5	6.000000	10.4	Antioxidant

Note: Sequence ID: unique identifier for each peptide or spectrum identified from the raw mass spectrometry data; −10 lgP: significance score of peptides identified through database search; Peptide: amino acid sequences of the identified peptides; Identified Bioactive Peptide Sequence: peptide sequences identified with antioxidant or neuroprotective activity; Similarity: amino acid match percentage of the identified bioactive peptide sequence against the peptide sequence in Pep-SH; Length: number of amino acid residues contained in the identified peptide; E-value: a statistical significance metric for BLAST alignment results—a smaller E-value indicates that the match is less likely to be random, and thus the result is more reliable; Bit Score: a quality score for BLAST alignment, reflecting the quality and significance of the sequence alignment—a higher score indicates a more reliable alignment; Putative Bioactivity: based on the similarity to known bioactive peptides, the potential bioactivity type of the identified peptide in Pep-SH is predicted.

**Table 6:** Representative neuroprotective peptides identified in Pep-SH by local BLAST search.

Sequence ID	−10 lgP	Peptide	Identified Bioactive Peptide Sequence	References	Similarity (%)	Length	E-Value	Bit Score	Bioactivity
314_A0A423SBQ1_PENVA_Mass=_895.4763_Length=_8	24.54	SLPQPVQQ	SLPSLPEPV	[33]	83.333	6	0.084	14.2	Neuroprotective
2609_A0A3R7PDJ9_PENVA_Mass=_496.2645_Length=_6	21.4	GPGLGP	EVSGPGLSPN	[34]	83.333	6	0.09	13.9	Neuroprotective
9739_A0A3R7PLR3_PENVA_Mass=_692.3381_Length=_6	9.99	LAFLDD	SLAFVDDVLN	[35]	83.333	6	0.09	13.9	Neuroprotective
8567_A0A423SR69_PENVA_Mass=_598.3326_Length=_6	13.84	SLPSVP	SLPSLPEPV	[33]	83.333	6	0.26	13.1	Neuroprotective
9589_A0A423T3E1_PENVA_Mass=_770.381_Length=_8	6.87	TPSTTPAP	HSMNPSTNPWHSTVHT	[36]	80	5	1.9	11.5	Neuroprotective
5229_A0A3R7LW47_PENVA_Mass=_687.3187_Length=_9	13.01	TGGGTAPAG	VLGGGSALLRSIPA	[37]	80	5	2.4	11.5	Neuroprotective
8923_A0A423T7B5_PENVA_Mass=_651.2976_Length=_6	7.67	TPSHNP	HSMNPSTNPWHSTVHT	[36]	80	5	3.3	11.2	Neuroprotective
11075_A0A3R7M2Q8_PENVA_Mass=_777.4021_Length=_9	5.06	VAPGPSPGP	EVSGPGLSPN	[36]	80	5	6.1	10.8	Neuroprotective
9083_A0A3R7QIH0_PENVA_Mass=_625.3071_Length=_7	7.5	AGPGVSP	EVSGPGLSPN	[36]	71.429	7	0.018	15	Neuroprotective
11017_A0A423S916_PENVA_Mass=_1150.587_Length=_12	5.13	PAAAAVPSLPEE	SLPSLPEPV	[36]	71.429	7	0.08	14.2	Neuroprotective
7548_A0A3R7QP52_PENVA_Mass=_756.3654_Length=_8	9.63	GLPSGPET	SLPSLPEPV	[36]	71.429	7	0.52	12.7	Neuroprotective
1447_A0A3R7QKI4_PENVA_Mass=_879.4338_Length=_8	19.54	PDLNEPVP	SLPSLPEPV	[36]	71.429	7	0.84	12.3	Neuroprotective
10199_A0A3R7QBU6_PENVA_Mass=_669.3486_Length=_6	6.07	TWGPIP	TWLPLPR	[36]	66.667	6	0.21	13.5	Neuroprotective
179_X2KWE4_PENVA_Mass=_818.381_Length=_7	16.23	DNLPPYT	NIPPLTQTPVVVPPFLQPE	[36]	66.667	6	0.36	13.1	Neuroprotective
2885_X2KWE4_PENVA_Mass=_703.3541_Length=_6	16.74	NLPPYT	NIPPLTQTPVVVPPFLQPE	[36]	66.667	6	0.36	12.7	Neuroprotective
3160_X2KWE4_PENVA_Mass=_946.476_Length=_8	16.23	KDNLPPYT	NIPPLTQTPVVVPPFLQPE	[36]	66.667	6	0.36	13.1	Neuroprotective
4646_A0A3R7M8C1_PENVA_Mass=_598.3326_Length=_6	13.84	SPVSLP	NAPVSIPQ	[36]	66.667	6	0.36	13.1	Neuroprotective
180_A0A423SBQ1_PENVA_Mass=_850.4548_Length=_8	27.06	SVPQPVQP	SLPSLPEPV	[36]	66.667	6	0.55	12.7	Neuroprotective
8568_A0A3R7M1Y2_PENVA_Mass=_598.3326_Length=_6	8.15	SIPSVP	SLPSLPEPV	[36]	66.667	6	0.59	12.7	Neuroprotective
6366_A0A423UA00_PENVA_Mass=_731.4581_Length=_7	11.37	PIVLPPP	NIPPLTQTPVVVPPFLQPE	[36]	66.667	6	0.79	12.3	Neuroprotective
2883_A0A423TLL3_PENVA_Mass=_615.2864_Length=_7	16.75	EGAGVSP	EVSGPGLSPN	[36]	66.667	6	0.86	12.3	Neuroprotective
3642_A0A3R7Q474_PENVA_Mass=_530.27_Length=_6	15.46	LLGGGD	VLGGGSALLRSIPA	[37]	66.667	6	1.6	11.5	Neuroprotective
5300_A0A3R7MDX3_PENVA_Mass=_1004.4927_Length=_11	12.93	GEQGPPGLPGP	SLPSLPEPV	[36]	66.667	6	2.3	11.9	Neuroprotective
10201_A0A423U3S0_PENVA_Mass=_926.4709_Length=_9	6.07	TPSNPLTTP	HSMNPSTNPWHSTVHT	[36]	66.667	6	2.9	11.2	Neuroprotective

**Table 7:** Predicted BBB permeability and toxicity of antioxidant peptides obtained by BLAST alignment.

Peptide	Similarity (%)	Mass	BBB Label	BBB+ Probability	Tox	Non-Tox
GDDAPR	100	629.2769	BBB+	0.655	0.262	0.738
GDGVTH	100	584.2554	BBB−	0.483	0.161	0.839
IIAPPE	100	638.3639	BBB+	0.587	0.192	0.808
DAYVGD	83.333	638.2547	BBB+	0.639	0.629	0.371
GDGVSH	83.333	570.2398	BBB−	0.494	0.146	0.854
GGLGGL	83.333	472.2645	BBB+	0.652	0.446	0.554
GPGSVGGP	83.333	626.3024	BBB−	0.487	0.027	0.973
GGFGGA	80	464.2019	BBB+	0.550	0.431	0.569

Note: Peptide: amino acid sequence of the identified peptide. Similarity (%): percentage of sequence identity between the identified peptide and a known bioactive peptide from the BLAST search. Mass: molecular weight of the peptide (Da). BBB Label: predicted blood–brain barrier permeability category; “BBB+” indicates a positive prediction for BBB penetration, while “BBB−” indicates a negative prediction. BBB+ Probability: predicted probability (ranging from 0 to 1) that the peptide can cross the BBB; higher values indicate greater likelihood. Tox: predicted probability of toxicity (0–1). Non-tox: predicted probability of non-toxicity (0–1); higher values indicate a safer profile.

**Table 8:** Predicted BBB permeability and toxicity of neuroprotective peptides identified by BLAST alignment.

Peptide	Similarity (%)	Mass	BBB Label	BBB+ Probability	Tox	Non-Tox
GPGLGP	83.333	496.2645	BBB+	0.681	0.085	0.915
SLPSVP	83.333	598.3326	BBB+	0.604	0.06	0.940
AGPGVSP	71.429	625.3071	BBB+	0.548	0.063	0.937
SPVSLP	66.667	598.3326	BBB+	0.612	0.061	0.939
SIPSVP	66.667	598.3326	BBB+	0.595	0.065	0.935
EGAGVSP	66.667	615.2864	BBB−	0.484	0.086	0.914
LLGGGD	66.667	530.27	BBB+	0.687	0.479	0.521

Note: Peptide: amino acid sequence of the identified peptide. Similarity (%): percentage of sequence identity between the identified peptide and a known bioactive peptide from the BLAST search. Mass: molecular weight of the peptide (Da). BBB Label: predicted blood–brain barrier permeability category; “BBB+” indicates a positive prediction for BBB penetration, while “BBB−” indicates a negative prediction. BBB+ Probability: predicted probability (ranging from 0 to 1) that the peptide can cross the BBB; higher values indicate greater likelihood. Tox: predicted probability of toxicity (0–1). Non-tox: predicted probability of non-toxicity (0–1); higher values indicate a safer profile.

## Data Availability

The raw MS data and peptide sequencing data are deposited in Baidu Netdisk with folder name ‘raw data for Jiawen Zhao’s shrimp head manuscript published on Foods’. The permanently valid linkage is https://pan.baidu.com/s/1Kry5PnSwg9mhR_agbMrB3Q (accessed on 27 May 2026) with the code of ‘1234’.

## Supplementary Materials

The following supporting information can be downloaded at: https://www.mdpi.com/article/10.3390/foods15111999/s1, Table S1. Annotation of major peaks in TIC chromatogram under positive ion mode. Table S2. Annotation of major peaks in TIC chromatogram under negative ion mode. Table S3. BLAST alignment results of Pep-SH with known antioxidant activity sequences. Table S4. BLAST alignment results of Pep-SH with known neuroprotective activity sequences.
