# Supplementary material for: Comprehensive Extraction of Shrimp Head Lipids and Peptides from Litopenaeus vannamei: Evaluation of Neuroprotective Potential and Constituent Analysis
Source: Foods. 2026 Jun 3;15(11):1999. doi: 10.3390/foods15111999 (PMC13256759; doi:10.3390/foods15111999)
Supplement: Supplementary file 1 [file foods-15-01999-s001.zip › foods-4319189-supplementary.pdf]

Table S1. Annotation of major peaks in TIC chromatogram under positive ion mode

Alignment ID	Average Rt(min)	Average Mz	Metabolite name	Formula	Adduct type	Total score	Dot product	Reverse dot product	Chemical structure
364	0.634	356.34668	NAE 20:0	$C_{22}H_{45}NO_2$	$[M+H]^+$	100	1.3	75	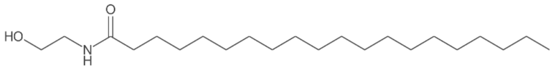
179	0.634	260.22943	SPB 14:1;3O	$C_{14}H_{29}NO_3$	$[M+H]^+$	100	0.6	87.5	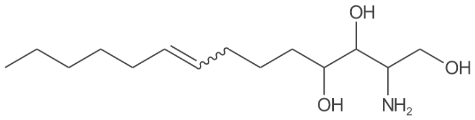
548	0.634	456.43829	SPB 28:1;3O	$C_{28}H_{57}NO_3$	$[M+H]^+$	100	0.1	83.3	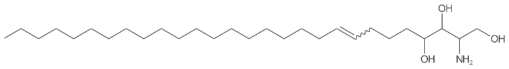

291	0.634	324.28131	NAE 18:2	$C_{20}H_{37}NO_2$	$[M+H]^+$	100	0.2	75	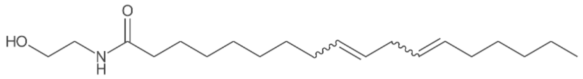
950	0.634	794.54962	HexCer 18:3;2O/24:6;O	$C_{48}H_{77}NO_9$	$[M+H-H_2O]^+$	100	0.2	81.2	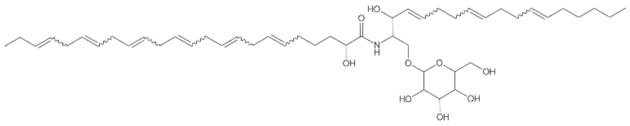
330	0.534	340.27634	Cer 12:1;2O/8:0;O	$C_{20}H_{39}NO_4$	$[M+H-H_2O]^+$	100	4.9	66.9	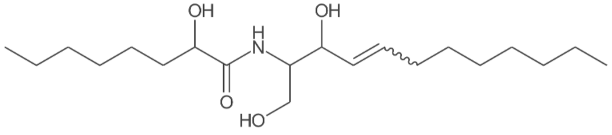
343	0.534	346.32584	SPB 20:0;3O	$C_{20}H_{43}NO_3$	$[M+H]^+$	100	null	89.8	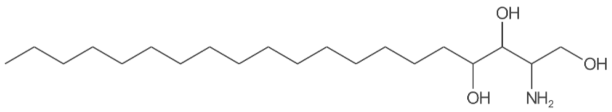

473	0.534	418.38504	MG 21:0	$C_{24}H_{48}O_4$	$[M+NH_4]^+$	100	null	53.7	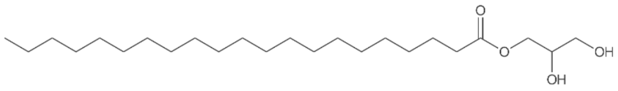
665	0.741	540.53564	Cer 25:0;2O/9:0	$C_{34}H_{69}NO_3$	$[M+H]^+$	100	null	65.8	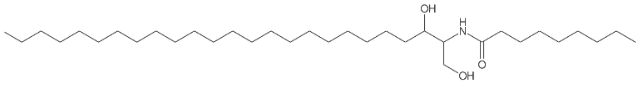
224	0.836	290.27673	SPB 16:0;3O	$C_{16}H_{35}NO_3$	$[M+H]^+$	100	null	95.7	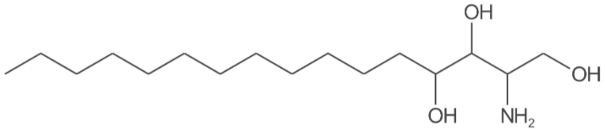
741	0.946	606.34583	w/o MS2:PS 8:0_15:2	$C_{29}H_{52}NO_{10}P$	$[M+H]^+$	100	null	null	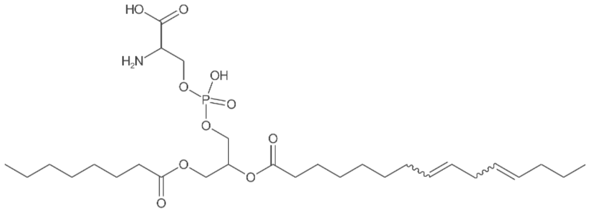

992	0.946	848.59308	TG 8:0_15:2_22:5;4O	$C_{48}H_{78}O_{11}$	$[M+NH_4]^+$	100	null	72.3	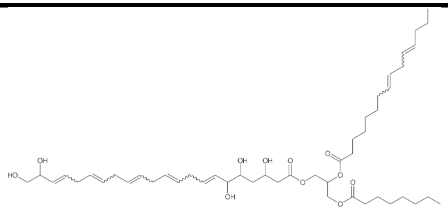
771	1.257	633.41034	SM 28:5;2O	$C_{33}H_{59}N_2O_6P$	$[M+Na]^+$	82.7	null	67.2	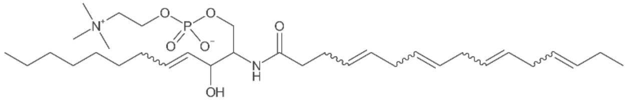
763	1.257	625.47363	DG 35:4	$C_{38}H_{66}O_5$	$[M+Na]^+$	92.2	null	75	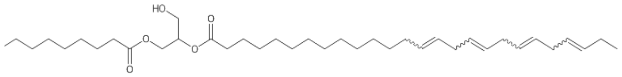
994	1.517	849.45258	PI 34:6	$C_{43}H_{71}O_{13}P$	$[M+Na]^+$	100	null	74.3	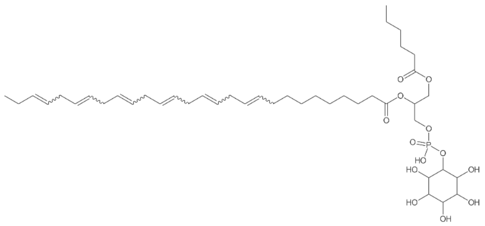

819	1.517	681.53827	DG 39:4	$C_{42}H_{74}O_5$	$[M+Na]^+$	96.1	null	75	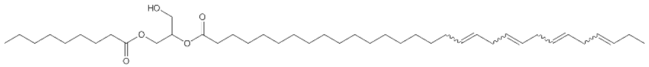
838	1.517	689.47498	TG 8:0_16:3_15:4	$C_{42}H_{66}O_6$	$[M+Na]^+$	100	null	63.5	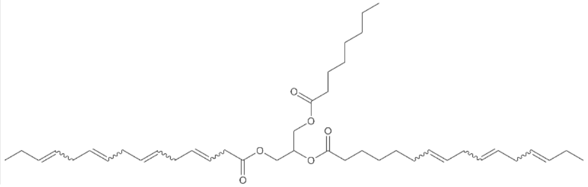
927	1.517	765.49353	SM 38:9;2O	$C_{43}H_{71}N_2O_6P$	$[M+Na]^+$	100	null	76.1	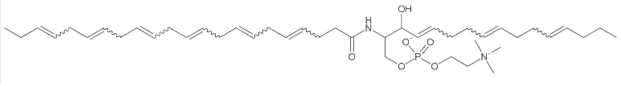
447	1.517	398.26047	w/o MS2:LPE O-13:0	$C_{18}H_{40}NO_6P$	$[M+H]^+$	100	null	null	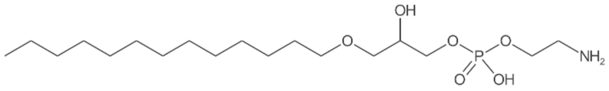

944	1.784	791.12909	Unknown	null	$[M+H]^+$	null	null	null	Unknown
73	2.191	190.99237	Unknown	null	$[M+H]^+$	null	null	null	Unknown
190	2.713	266.99179	Unknown	null	$[M+Na]^+$	null	null	null	Unknown
895	3.054	736.54199	HexCer 18:2;3O/18:2;(2OH)	$C_{42}H_{75}NO_{10}$	$[M+H-H_2O]^+$	100	null	97.1	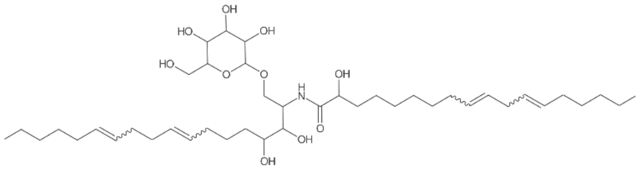

885	3.054	721.51349	SL 15:2;O/26:5;O	$C_{41}H_{69}NO_6S$	$[M+NH_4]^+$	100	13.9	57.7	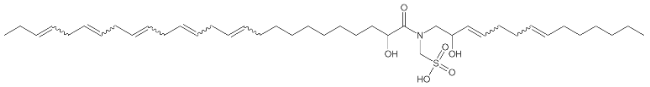
860	3.054	706.50171	w/o MS2:SL 21:3;O/20:3;O	$C_{41}H_{71}NO_6S$	$[M+H]^+$	100	null	null	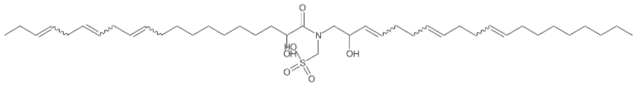
828	3.054	685.54614	TG 10:0_10:0_18:2	$C_{41}H_{74}O_6$	$[M+Na]^+$	100	1.1	73.7	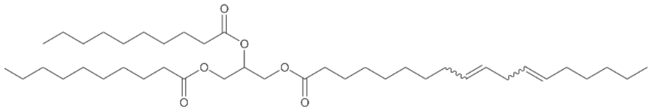
1091	3.054	1364.0091	PI 71:8	$C_{80}H_{141}O_{13}P$	$[M+Na]^+$	100	null	88.4	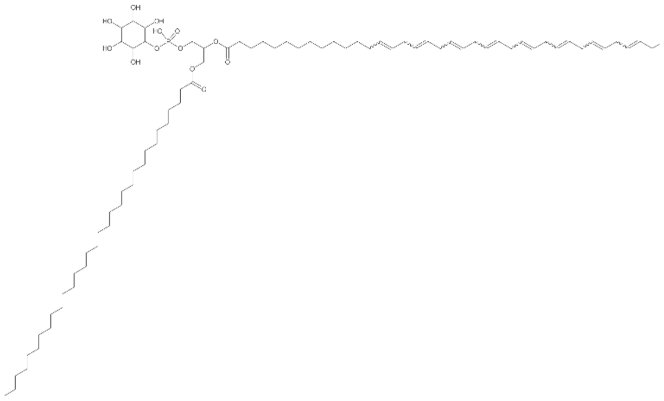

821	3.388	684.20874	w/o MS2:RIKEN P-VS1 ID-7591 from Cell_HEK293_WT_N_Ctr	null	$[M+H]^+$	73.5	null	null	Unknown

Note: Alignment ID: unique identifier for each feature peak alignment result across analyses; Average Rt(min): mean retention time in minutes, indicating the average elution time of the metabolite on the column; Average Mz: mean mass-to-charge ratio in Daltons, representing the detected average m/z of the feature peak; Metabolite name: compound name obtained by matching against a database; Formula: chemical molecular formula of the metabolite; Adduct type: the ionic form detected in mass spectrometry (e.g., $[M+H]^+$, $[M-H]^-$); Total score: comprehensive score evaluating the confidence of metabolite annotation (higher score, up to 100, indicates more reliable match); Dot product: similarity measure between the experimental MS/MS spectrum and the database reference spectrum (higher value means greater similarity; null indicates no MS/MS data available); Reverse dot product: similarity metric that does not penalize database-absent fragment ions, suitable for complex backgrounds (higher value indicates better match; null indicates no such data).

Table S2. Annotation of major peaks in TIC chromatogram under negative ion mode

Alignment ID	Average Rt(min)	Average Mz	Metabolite name	Formula	Adduct type	Total score	Dot product	Reverse dot product	Chemical structure
361	0.636	465.30469	AAHFA 15:4/15:4;O	$C_{30}H_{42}O_4$	$[M-H]^-$	100	31.8	82.8	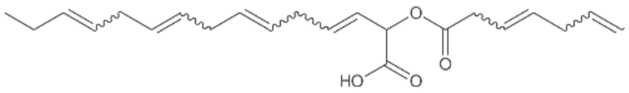

400	0.636	485.2825 9	w/o MS2:RIKE N N-VS1 ID-2081 from Mouse_Fe ces_WT_N _Ctr	null	[M+Br] -	71.2	null	null	Unknown
166	0.755	341.2005	FA 18:3;4O	$C_{18}H_{30}O_6$	$[M-H]^-$	100	0.8	75	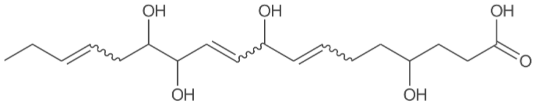
492	0.755	550.2840 6	PS 6:0_13:1	$C_{25}H_{46}NO_{10}P$	$[M-H]^-$	100	0.7	51.6	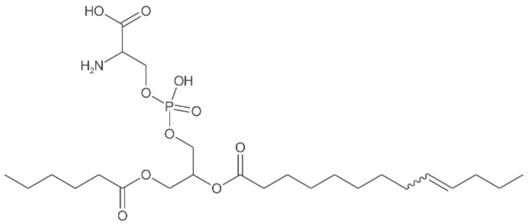
81	0.932	271.2282 4	FA 16:0;1O	$C_{16}H_{32}O_3$	$[M-H]^-$	100	0.9	75	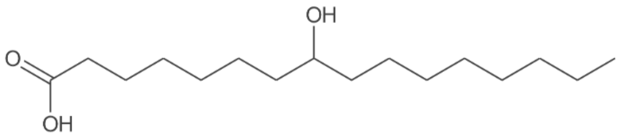

590	0.932	655.42554	NAGlySer 18:4;O(FA 15:2)	$C_{38}H_{60}N_2O_7$	$[M-H]^-$	100	0.3	85.3	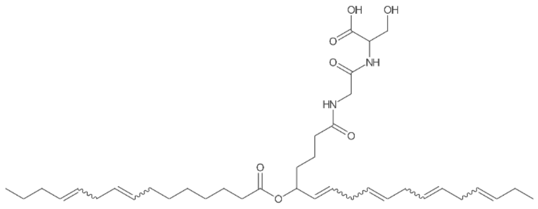
399	1.251	485.28229	w/o MS2:RIKEN N-VS1 ID-2080 from Mouse_Feces_WT_N_Ctr	null	$[M-H]^-$	69.4	null	null	Unknown
248	1.441	401.16199	w/o MS2:RIKEN N-VS1 ID-1467 from Mouse_Plasma_ApoEKO_N_F1	null	$[M+FA-H]^-$	70.6	null	null	Unknown
282	1.441	417.30142	AAHFA 18:4/8:0;O	$C_{26}H_{42}O_4$	$[M-H]^-$	100	0.3	95.3	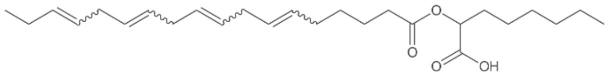

593	1.441	656.39447	PE 20:4_9:0;1O	$C_{34}H_{60}NO_9P$	$[M-H]^-$	100	0.1	97.7	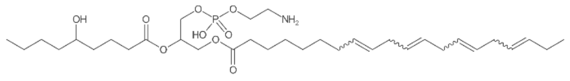
427	1.501	502.22333	w/o MS2:LPS 17:4	$C_{23}H_{38}NO_9P$	$[M-H]^-$	98.1	null	null	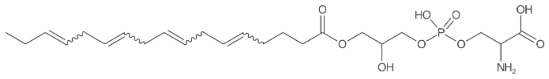
92	1.501	283.26291	FA 18:0	$C_{18}H_{36}O_2$	$[M-H]^-$	100	3.7	75	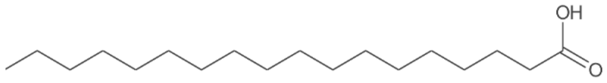
179	1.758	351.24875	AAHFA 5:0/16:2;O	$C_{21}H_{36}O_4$	$[M-H]^-$	100	0.4	94	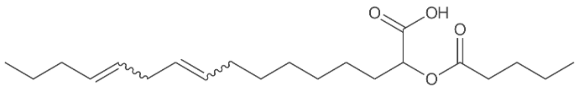

Note: Alignment ID: unique identifier for each feature peak alignment result across analyses; Average Rt(min): mean retention time in minutes, indicating the average elution time of the metabolite on the column; Average Mz: mean mass-to-charge ratio in Daltons, representing the detected average m/z of the feature peak; Metabolite name: compound name obtained by matching against a database; Formula: chemical molecular formula of the metabolite; Adduct type: the ionic form detected in mass spectrometry (e.g., $[M+H]^+$, $[M-H]^-$); Total score: comprehensive score evaluating the confidence of metabolite annotation (higher score, up to 100, indicates more reliable match); Dot product: similarity measure between the experimental MS/MS spectrum and the database reference spectrum (higher value means greater similarity; null indicates no MS/MS data available); Reverse dot product: similarity metric that does not penalize database-absent fragment ions, suitable for complex backgrounds (higher value indicates better match; null indicates no such data).

Table S3. BLAST alignment results of Pep-SH with known antioxidant activity sequences

Sequence ID	Peptide	Identified Bioactive Peptide Sequence	Similarity (%)	Length	E-value	Bit Score
50_A0A423SEE9_PENVA_Mass=_786.3144_Length=_8	DSGDGVTH	DSGDGVTHTVPIYEG	100	8	0.000301	19.6
10574_A0A423T7C2_PENVA_Mass=_922.56_Length=_8	IIAPPERK	KIKIIAPPERKYSVW	100	8	0.000351	19.2
1149_A0A3R7PEZ1_PENVA_Mass=_777.3908_Length=_7	TVPIYEG	DSGDGVTHTVPIYEG	100	7	0.000776	18.1
190_A0A423T7C2_PENVA_Mass=_700.314_Length=_7	AGDDAPR	AGDDAPRAVF	100	7	0.001000	17.7
675_A0A423T7C2_PENVA_Mass=_904.4039_Length=_9	GFAGDDAPR	AGDDAPRAVF	100	7	0.001000	17.7
2962_A0A423T7C2_PENVA_Mass=_847.3824_Length=_8	FAGDDAPR	AGDDAPRAVF	100	7	0.001000	17.7
6322_A0A423SEE9_PENVA_Mass=_671.2874_Length=_7	SGDGVTH	DSGDGVTHTVPIYEG	100	7	0.001000	17.7
161_A0A3R7PEZ1_PENVA_Mass=_720.3694_Length=_6	TVPIYE	DSGDGVTHTVPIYEG	100	6	0.003000	16.2
1306_A0A423T7C2_PENVA_Mass=_629.2769_Length=_6	GDDAPR	AGDDAPRAVF	100	6	0.003000	16.5
356_A0A423SEE9_PENVA_Mass=_584.2554_Length=_6	GDGVTH	DSGDGVTHTVPIYEG	100	6	0.004000	16.2
10819_A0A423TWP3_PENVA_Mass=_949.4981_Length=_9	TGGLARYVN	PGGVGGLARYT	100	6	0.006000	15.8
2334_A0A423T7C2_PENVA_Mass=_638.3639_Length=_6	IIAPPE	KIKIIAPPERKYSVW	100	6	0.007000	15.4
7_A0A423T7C2_PENVA_Mass=_1031.3978_Length=_10	VCDSGDGVSH	DSGDGVTHTVPIYEG	87.5	8	0.000810	18.5
162_A0A3R7SUV3_PENVA_Mass=_720.3694_Length=_6	TVPLYE	DSGDGVTHTVPIYEG	83.333	6	0.006000	15.4
3120_A0A423T7C2_PENVA_Mass=_638.2547_Length=_6	DAYVGD	VGMGQKDSYVGDEAQSKRGILT	83.333	6	0.012000	15
888_A0A423T7C2_PENVA_Mass=_570.2398_Length=_6	GDGVSH	DSGDGVTHTVPIYEG	83.333	6	0.014000	15
2588_A0A3R7PTG0_PENVA_Mass=_774.3508_Length=_7	DRDGVVD	AVDINRDGVVSE	83.333	6	0.085000	13.9
2588_A0A3R7PTG0_PENVA_Mass=_774.3508_Length=_7	DRDGVVD	VDINRDGVVSE	83.333	6	0.097000	13.9
5216_A0A3R7MI39_PENVA_Mass=_472.2645_Length=_6	GGLGGL	PGGVGGLARYT	83.333	6	0.480000	12.7

Continued Table S3.BLAST alignment results of Pep-SH with known antioxidant activity sequences

7311_A0A3R7PG97_PENVA _Mass=_626.3024_Length=_8	GPGSVGGP	PGGVGGLA RYT	83.333	6	0.510 000	12. 7
8819_A0A3R7MTS3_PENVA _Mass=_653.3384_Length=_6	TPDPKP	DPALATEPD PMPF	80	5	0.890 000	11. 9
3440_A0A3R7LZ42_PENVA_ Mass=_464.2019_Length=_6	GGFGGA	PGGVGGLA RYT	80	5	6.000 000	10. 4
7792_A0A3R7LXZ4_PENVA _Mass=_858.397_Length=_8	EVATEVDP	DPALATEPD PMPF	71.429	7	0.095 000	13. 9
9819_A0A423TG17_PENVA_ Mass=_827.3926_Length=_8	TPGNFVGH	FDTPSDFVK	66.667	6	0.120 000	13. 9
3863_A0A423TMZ4_PENVA _Mass=_578.2812_Length=_6	TPHGAP	LADQSRNP HSAP	66.667	6	0.140 000	13. 5
7340_A0A423SBQ1_PENVA_ Mass=_470.2489_Length=_6	AVGAGP	AIGVGPEVN QSQL	66.667	6	0.140 000	13. 5
8966_A0A3R7QR84_PENVA _Mass=_685.3282_Length=_7	VAGEDVP	AGDDAPRA VF	66.667	6	0.170 000	13. 5
79_A0A423TS87_PENVA_M ass=_2190.9343_Length=_22	MICAGVPE GGKDSCQG DSGGPL	VGMGQKDS YVGDEAQS KRGILT	66.667	9	0.190 000	13. 9
146_A0A423TS87_PENVA_ Mass=_1786.7791_Length=_1 9	AGVPEGGK DSCQGDSG GPL	VGMGQKDS YVGDEAQS KRGILT	66.667	9	0.200 000	13. 9
110_A0A423TS87_PENVA_ Mass=_1715.742_Length=_18	GVPEGGKD SCQGDSGG PL	VGMGQKDS YVGDEAQS KRGILT	66.667	9	0.210 000	13. 9
5403_A0A3R7MP46_PENVA _Mass=_680.303_Length=_6	PAYGHH	DPGYMHHK FAIV	66.667	6	0.270 000	13. 1
10382_A0A3R7PUT9_PENV A_Mass=_929.4494_Length=_ 9	TPSPTPFSP	DPALATEPD PMPF	66.667	6	0.540 000	12. 7
5215_A0A3R7PW33_PENVA _Mass=_472.2645_Length=_6	GGIGGI	PGGVGGLA RYT	66.667	6	0.550 000	12. 3
9862_A0A3R7PJP8_PENVA_ Mass=_719.3966_Length=_7	TVPHAVP	DSGDGVTH TVPIYEG	66.667	6	0.740 000	12. 3
4057_A0A423TSE5_PENVA_ Mass=_696.2966_Length=_6	EPEPEP	DPALATEPD PMPF	66.667	6	0.970 000	11. 9
5627_A0A423SF08_PENVA_ Mass=_645.3082_Length=_8	AGGTGGV Q	PGGVGGLA RYT	66.667	6	2.700 000	11. 2
9979_A0A423TB54_PENVA_ Mass=_826.3344_Length=_8	DGPDPETP	EPVPERPVK	66.667	6	3.100 000	11. 2
8467_A0A3R7PCW4_PENVA _Mass=_1027.5087_Length=_ 10	RPDLPPGY GG	PGGVGGLA RYT	66.667	6	6.200 000	10. 8
4562_A0A3R7QEX8_PENVA _Mass=_859.4552_Length=_8	SAPPPPRP	EPVPERPVK	66.667	6	7.300 000	10. 4
9909_A0A423UB02_PENVA_ Mass=_908.4967_Length=_10	TGATAPVP VP	DPALATEPD PMPF	62.5	8	0.280 000	13. 1
9768_A0A423T1P4_PENVA_ Mass=_867.5065_Length=_8	SLKVIDPP	KIKIIAPPER KYSVW	57.143	7	0.120 000	13. 5
171_A0A423T7C2_PENVA_ Mass=_665.3496_Length=_7	SGPGIVH	DSGDGVTH TVPIYEG	57.143	7	0.200 000	13. 1
10195_A0A423SAL8_PENVA _Mass=_683.2697_Length=_7	SPGGMGH	DPGYMHHK FAIV	57.143	7	0.610 000	12. 3
5647_A0A3R7MMX9_PENV A_Mass=_897.576_Length=_8	SLAPVKRK	KIKIIAPPER KYSVW	57.143	7	0.750 000	12. 3
5007_A0A423TF99_PENVA_ Mass=_766.4225_Length=_7	VEPKPTP	DPALATEPD PMPF	57.143	7	0.860 000	11. 9
4440_A0A423TF99_PENVA_ Mass=_895.4651_Length=_8	VEPKPTPE	DPALATEPD PMPF	57.143	7	1.000 000	11. 9
10142_A0A423TF99_PENVA Mass=_1024.5077_Length=_9	VEPKPTPEE	DPALATEPD PMPF	57.143	7	1.200 000	11. 9

Continued Table S3.BLAST alignment results of Pep-SH with known antioxidant activity sequences

10322_A0A3R7PI37_PENVA_Mass=_737.3708_Length=_7	TGDLVPH	DSGDGVTHTVPIYEG	57.143	7	1.300000	11.5
5559_A0A423TYK3_PENVA_Mass=_794.381_Length=_8	TAPEPSPP	DPALATEPDPMPF	57.143	7	1.500000	11.5
8593_A0A423SE33_PENVA_Mass=_878.5086_Length=_8	VSPRPARP	EPVPERPVK	57.143	7	2.200000	11.5
9715_A0A3R7LW14_PENVA_Mass=_818.4174_Length=_8	PEPPPSPV	DPALATEPDPMPF	57.143	7	5.500000	10.4
7700_A0A3R7SMJ4_PENVA_Mass=_909.4708_Length=_9	TPPHPAPPP	DPALATEPDPMPF	57.143	7	6.000000	10.4
313_A0A423TIS4_PENVA_Mass=_890.4134_Length=_8	HTDPIPDP	DPALATEPDPMPF	57.143	7	9.100000	10
3824_A0A423TIS4_PENVA_Mass=_881.4494_Length=_8	TDPIPDPK	DPALATEPDPMPF	57.143	7	9.200000	10
1928_A0A423TIS4_PENVA_Mass=_753.3545_Length=_7	TDPIPDP	DPALATEPDPMPF	57.143	7	9.400000	10
5901_A0A3R7LY93_PENVA_Mass=_880.429_Length=_10	LGDGPPGTAP	DSGDGVTHTVPIYEG	55.556	9	0.270000	13.1
38_A0A3R7MR58_PENVA_Mass=_1333.5568_Length=_14	GGKDSCQGDSGGPL	VGMGQKDSYVGDEAQSKRGILT	54.545	11	0.160000	13.9
173_A0A423T7C2_PENVA_Mass=_794.3922_Length=_8	ESGPGIVH	DSGDGVTHTVPIYEG	50	8	0.077000	13.9
74_A0A3R7N633_PENVA_Mass=_923.4348_Length=_9	EESGPGIVH	DSGDGVTHTVPIYEG	50	8	0.084000	13.9
587_A0A3R7M3B1_PENVA_Mass=_908.4352_Length=_9	NESGPGIVH	DSGDGVTHTVPIYEG	50	8	0.092000	13.9
76_A0A423T7C2_PENVA_Mass=_1072.4825_Length=_10	YDESGPGIVH	DSGDGVTHTVPIYEG	50	8	0.140000	13.5
24_A0A423T7C2_PENVA_Mass=_909.4192_Length=_9	DESGPGIVH	DSGDGVTHTVPIYEG	50	8	0.150000	13.5
8024_A0A423T0F7_PENVA_Mass=_1137.5778_Length=_11	TAQNPSQAPKP	LADQSRNPHSAP	50	8	0.460000	12.7
17_A0A423T5F8_PENVA_Mass=_1509.6735_Length=_14	WSEPIDDGGSPITH	DSGDGVTHTVPIYEG	50	8	0.680000	12.7
60_A0A423T5F8_PENVA_Mass=_1323.5942_Length=_13	SEPIDDGGSPITH	DSGDGVTHTVPIYEG	50	8	0.720000	12.3
9909_A0A423UB02_PENVA_Mass=_908.4967_Length=_10	TGATAPVPVP	DSGDGVTHTVPIYEG	50	8	0.810000	12.3
564_A0A423T5F8_PENVA_Mass=_853.393_Length=_9	SDGGAPITH	DSGDGVTHTVPIYEG	50	8	1.000000	11.9
1053_A0A423T7C2_PENVA_Mass=_578.3176_Length=_6	GPGIVH	DSGDGVTHTVPIYEG	50	6	1.300000	11.5
96_A0A423T5F8_PENVA_Mass=_1151.5458_Length=_12	AGPGDPSEPTKP	DPALATEPDPMPF	50	8	2.400000	11.5
1311_A0A423T5F8_PENVA_Mass=_1279.6408_Length=_13	KAGPGDPSEPTKP	DPALATEPDPMPF	50	8	2.400000	11.5
52_A0A423T5F8_PENVA_Mass=_1288.6047_Length=_13	AGPGDPSEPTKPH	DPALATEPDPMPF	50	8	2.500000	11.5
2_A0A423T5T3_PENVA_Mass=_2128.1415_Length=_20	GEKPPSIKPEEPIEGPVTKP	EPVPERPVK	50	6	6.300000	11.2
30_A0A3R7NSY1_PENVA_Mass=_1164.5186_Length=_10	IEPEPEPEPE	DPALATEPDPMPF	44.444	9	1.400000	11.9
9771_A0A3R7QP72_PENVA_Mass=_1215.6322_Length=_11	TPCLQIPPPPP	DPALATEPDPMPF	44.444	9	8.900000	10.4

Continued Table S3.BLAST alignment results of Pep-SH with known antioxidant activity sequences

10889_A0A3R7MR41_PENVA_Mass=_930.3871_Length=_10	SGGFGGFGGF	PGGVGGLARYT	40	10	2.000000	11.5
10099_A0A3R7QDL5_PENVA_Mass=_1128.6542_Length=_12	PSLVPAAAPLPP	DPALATEPDPMPF	36.364	11	9.600000	10.4

Note: Sequence ID : unique identifier for each peptide or spectrum identified from the raw mass spectrometry data; Peptide: amino acid sequences of the identified peptides; Identified Bioactive Peptide Sequence: peptide sequences identified with antioxidant or neuroprotective activity; Similarity: amino acid match percentage of the identified bioactive peptide sequence against the peptide sequence in Pep-SH; Length: number of amino acid residues contained in the identified peptide; E-value: a statistical significance metric for BLAST alignment results, a smaller E-value indicates that the match is less likely to be random, and thus the result is more reliable; Bit Score: a quality score for BLAST alignment, reflecting the quality and significance of the sequence alignment, a higher score indicates a more reliable alignment.

Table S4.BLAST alignment results of Pep-SH with known neuroprotective activity sequences

Sequence ID	Peptide	Identified Bioactive Peptide Sequence	Similarity (%)	Length	E-value	Bit Score
314_A0A423SBQ1_PENVA_Mass=_895.4763_Length=_8	SLPQPVQQ	SLPSLPEPV	83.333	6	0.084	14.2
2609_A0A3R7PDJ9_PENVA_Mass=_496.2645_Length=_6	GPGLGP	EVSGPGLSPN	83.333	6	0.09	13.9
9739_A0A3R7PLR3_PENVA_Mass=_692.3381_Length=_6	LAFLDD	SLAFVDDVLN	83.333	6	0.09	13.9
8567_A0A423SR69_PENVA_Mass=_598.3326_Length=_6	SLPSVP	SLPSLPEPV	83.333	6	0.26	13.1
9589_A0A423T3E1_PENVA_Mass=_770.381_Length=_8	TPSTTPAP	HSMNPSTNPWHSTVHT	80	5	1.9	11.5
5229_A0A3R7LW47_PENVA_Mass=_687.3187_Length=_9	TGGGTAPAG	VLGGGSALLRSIPA	80	5	2.4	11.5
8923_A0A423T7B5_PENVA_Mass=_651.2976_Length=_6	TPSHNP	HSMNPSTNPWHSTVHT	80	5	3.3	11.2
11075_A0A3R7M2Q8_PENVA_Mass=_777.4021_Length=_9	VAPGPSPGP	EVSGPGLSPN	80	5	6.1	10.8
9083_A0A3R7QIH0_PENVA_Mass=_625.3071_Length=_7	AGPGVSP	EVSGPGLSPN	71.429	7	0.018	15
11017_A0A423S916_PENVA_Mass=_1150.587_Length=_12	PAAAAVPSLPEE	SLPSLPEPV	71.429	7	0.08	14.2
7548_A0A3R7QP52_PENVA_Mass=_756.3654_Length=_8	GLPSGPET	SLPSLPEPV	71.429	7	0.52	12.7
1447_A0A3R7QKI4_PENVA_Mass=_879.4338_Length=_8	PDLNEPVP	SLPSLPEPV	71.429	7	0.84	12.3
10199_A0A3R7QBU6_PENVA_Mass=_669.3486_Length=_6	TWGPIP	TWLPLPR	66.667	6	0.21	13.5
179_X2KWE4_PENVA_Mass=_818.381_Length=_7	DNLPPYT	NIPPLTQTPVVVPPFLQPE	66.667	6	0.36	13.1
2885_X2KWE4_PENVA_Mass=_703.3541_Length=_6	NLPPYT	NIPPLTQTPVVVPPFLQPE	66.667	6	0.36	12.7

Continued Table S4. BLAST alignment results of Pep-SH with known neuroprotective activity sequences

3160_X2KWE4_PENVA_Mass=_946.476_Length=_8	KDNLPPYT	NIPPLTQTPVVVPPFLQPE	66.667	6	0.36	13.1
4646_A0A3R7M8C1_PENVA_Mass=_598.3326_Length=_6	SPVSLP	NAPVSIPQ	66.667	6	0.36	13.1
180_A0A423SBQ1_PENVA_Mass=_850.4548_Length=_8	SVPQPVQP	SLPSLPEPV	66.667	6	0.55	12.7
8568_A0A3R7M1Y2_PENVA_Mass=_598.3326_Length=_6	SIPSVP	SLPSLPEPV	66.667	6	0.59	12.7
6366_A0A423UA00_PENVA_Mass=_731.4581_Length=_7	PIVLPPP	NIPPLTQTPVVVPPFLQPE	66.667	6	0.79	12.3
2883_A0A423TLL3_PENVA_Mass=_615.2864_Length=_7	EGAGVSP	EVSGPGLSPN	66.667	6	0.86	12.3
3642_A0A3R7Q474_PENVA_Mass=_530.27_Length=_6	LLGGGD	VLGGGSALLRSIPA	66.667	6	1.6	11.5
5300_A0A3R7MDX3_PENVA_Mass=_1004.4927_Length=_11	GEQGPPGLPGP	SLPSLPEPV	66.667	6	2.3	11.9
10201_A0A423U3S0_PENVA_Mass=_926.4709_Length=_9	TPSNPLTTP	HSMNPSTNPWHSTVHT	66.667	6	2.9	11.2
10628_A0A423TPW6_PENVA_Mass=_844.3926_Length=_10	TPAGTTGGPS	CARLNCVPKGTSGNTETCPCYASLHSCRKYG	57.143	7	0.61	12.7
7537_A0A423U5W2_PENVA_Mass=_1080.5815_Length=_12	GSPISIPVGPGT	NAPVSIPQ	57.143	7	0.26	13.5
7573_A0A3R7LXR8_PENVA_Mass=_711.3075_Length=_7	GSPISIPVGPGT	HSMNPSTNPWHSTVHT	57.143	7	0.79	12.3
9894_A0A3R7PBU7_PENVA_Mass=_1019.54_Length=_10	TPPPASRTPP	NIPPLTQTPVVVPPFLQPE	57.143	7	0.89	12.3
2_A0A423T5T3_PENVA_Mass=_2128.1415_Length=_20	GEKPPSIKPEEPIEGPVTKP	NIPPLTQTPVVVPPFLQPE	57.143	7	0.97	12.7
9551_A0A423STH8_PENVA_Mass=_755.4217_Length=_7	IPSFPVP	SLPSLPEPV	57.143	7	0.97	12.3
9660_A0A423TD75_PENVA_Mass=_972.4804_Length=_8	ELPFIPEE	SLPSLPEPV	57.143	7	1.4	11.9
8862_A0A423TYP7_PENVA_Mass=_795.3875_Length=_9	PGGGIGNQP	CARLNCVPKGTSGNTETCPCYASLHSCRKYG	57.143	7	2.3	11.2
8760_A0A3R7QMT7_PENVA_Mass=_1008.4876_Length=_10	TSPSHPTSVP	HSMNPSTNPWHSTVHT	57.143	7	3.4	11.2
10747_A0A423UB90_PENVA_Mass=_1059.5964_Length=_10	PLTPTLQPPP	NIPPLTQTPVVVPPFLQPE	57.143	7	4.3	10.8
8313_A0A3R7MA17_PENVA_Mass=_993.5131_Length=_9	TPPKEQPTP	NIPPLTQTPVVVPPFLQPE	57.143	7	6.4	10.4
4607_A0A423TSL2_PENVA_Mass=_1027.4822_Length=_11	PGVSSPSVSPD	EVSGPGLSPN	55.556	9	0.13	13.9
3786_A0A3R7QHJ7_PENVA_Mass=_1312.5692_Length=_11	PICNPVNCPWG	HSMNPSTNPWHSTVHT	50	8	0.38	13.1

Continued Table S4. BLAST alignment results of Pep-SH with known neuroprotective activity sequences

9433_A0A3R7QUT3_PENVA_Mass=_831.4338_Length=_9	ASGSSLTLP	CARLNCVPKGTSGNTETCPCYASLHSCRKYG	50	8	0.67	12.3
8837_A0A3R7Q0B0_PENVA_Mass=_1019.5763_Length=_9	TLPDRPKPP	SLPSLPEPV	50	8	0.86	12.3
9343_A0A3R7PFF0_PENVA_Mass=_878.4102_Length=_8	GGCGKLKC	CARLNCVPKGTSGNTETCPCYASLHSCRKYG	50	6	0.88	12.3
3644_A0A423SCY3_PENVA_Mass=_530.27_Length=_6	IIGGGD	VLGGGSALLRSIPA	50	6	1.6	11.5
28_X2KWE4_PENVA_Mass=_1365.6776_Length=_13	ESSVTVPDVPSIH	CARLNCVPKGTSGNTETCPCYASLHSCRKYG	44.444	9	0.23	13.5
9_X2KWE4_PENVA_Mass=_1149.603_Length=_11	SVTVPDVPSIH	CARLNCVPKGTSGNTETCPCYASLHSCRKYG	44.444	9	0.25	13.5
199_X2KWE4_PENVA_Mass=_1191.6135_Length=_11	SVTVPDVPSIH	CARLNCVPKGTSGNTETCPCYASLHSCRKYG	44.444	9	0.25	13.5
25_X2KWE4_PENVA_Mass=_1278.6456_Length=_12	SSVTVPDVPSIH	CARLNCVPKGTSGNTETCPCYASLHSCRKYG	44.444	9	0.26	13.5
56_X2KWE4_PENVA_Mass=_1553.7573_Length=_15	STESSVTVPDVPSIH	CARLNCVPKGTSGNTETCPCYASLHSCRKYG	44.444	9	0.27	13.5
29_X2KWE4_PENVA_Mass=_1236.635_Length=_12	SSVTVPDVPSIH	CARLNCVPKGTSGNTETCPCYASLHSCRKYG	44.444	9	0.26	13.5
40_X2KWE4_PENVA_Mass=_963.5025_Length=_9	TVPDVPSIH	CARLNCVPKGTSGNTETCPCYASLHSCRKYG	44.444	9	0.7	12.3
22_X2KWE4_PENVA_Mass=_1062.5709_Length=_10	VTVPDVPSIH	CARLNCVPKGTSGNTETCPCYASLHSCRKYG	44.444	9	0.75	12.3
5278_X2KWE4_PENVA_Mass=_1078.5295_Length=_10	TVPDVPSIHD	CARLNCVPKGTSGNTETCPCYASLHSCRKYG	44.444	9	0.75	12.3
6_A0A423SXN9_PENVA_Mass=_1922.868_Length=_17	VTVPIVSDDECRDAYGQ	CARLNCVPKGTSGNTETCPCYASLHSCRKYG	43.75	16	6.9	10.8
21_A0A423SXN9_PENVA_Mass=_2179.0215_Length=_19	QKVTVPIVSDDECRDAYGQ	CARLNCVPKGTSGNTETCPCYASLHSCRKYG	43.75	16	7.1	10.8
1_A0A423SXN9_PENVA_Mass=_2050.9629_Length=_18	KVTVPIVSDDECRDAYGQ	CARLNCVPKGTSGNTETCPCYASLHSCRKYG	43.75	16	7.3	10.8

Continued Table S4. BLAST alignment results of Pep-SH with known neuroprotective activity sequences

7417_X2KWE4_PENVA_Mass=_1177.5979_Length=_11	VTVPDVPSIHD	CARLNCVPKGTSGNTETCPCYASLHSCRKYG	40	10	0.16	13.9
104_X2KWE4_PENVA_Mass=_1264.6299_Length=_12	SVTVPDVPSIHD	CARLNCVPKGTSGNTETCPCYASLHSCRKYG	40	10	0.18	13.9
11124_A0A423TY16_PENVA_Mass=_1694.845_Length=_16	TVPPPPSTPPKCPSHP	NIPPLTQTPVVVPPFLQPE	38.462	13	7	10.8
2185_X2KWE4_PENVA_Mass=_1377.714_Length=_13	SVTVPDVPSIHDL	CARLNCVPKGTSGNTETCPCYASLHSCRKYG	36.364	11	0.16	13.9

Note: Sequence ID : unique identifier for each peptide or spectrum identified from the raw mass spectrometry data; Peptide: amino acid sequences of the identified peptides; Identified Bioactive Peptide Sequence: peptide sequences identified with antioxidant or neuroprotective activity; Similarity: amino acid match percentage of the identified bioactive peptide sequence against the peptide sequence in Pep-SH; Length: number of amino acid residues contained in the identified peptide; E-value: a statistical significance metric for BLAST alignment results, a smaller E-value indicates that the match is less likely to be random, and thus the result is more reliable; Bit Score: a quality score for BLAST alignment, reflecting the quality and significance of the sequence alignment, a higher score indicates a more reliable alignment.
